# Development and preliminary evaluation of an AI-enhanced three-dimensional integrated quality model for quality-sensitive indicators in operating room management: a prospective single-center study

**DOI:** 10.3389/fmed.2026.1830302

**Published:** 2026-05-26

**Authors:** Luping Li, Jianshu Cai, Xiaoling Huang, Jing Cai

**Affiliations:** Nursing Department, Sir Run Run Shaw Hospital, Zhejiang University School of Medicine, Hangzhou, China

**Keywords:** artificial intelligence, digital transformation, machine learning, nursing informatics, operating room efficiency, predictive maintenance, quality indicators

## Abstract

**Background:**

Traditional quality measurement systems in operating rooms often fail to capture the interdependence among medical equipment performance, operational efficiency, and staff effectiveness. Artificial intelligence and machine learning technologies may strengthen quality-monitoring frameworks when they are embedded within clearly defined operational protocols and human workflow support.

**Objective:**

To develop and preliminarily evaluate an artificial intelligence-enhanced three-dimensional integrated quality model for quality-sensitive indicators that integrates real-time equipment monitoring, predictive analytics, and staff performance metrics within an operating room (OR) quality-management intervention.

**Methods:**

A prospective single-center study was conducted at Sir Run Run Shaw Hospital, Zhejiang University School of Medicine, Hangzhou, China (*n* = 347 equipment units, 156 OR staff) from January 2024 to December 2024. The study used a pragmatic pre-post implementation design in which an AI-enhanced three-dimensional integrated quality model was implemented together with structured staff training, protocol reinforcement, and workflow optimization. Primary outcomes included equipment utilization efficiency, operational performance metrics, and staff productivity indices measured through IoT sensors, electronic health records, and validated assessment tools, with analyses specified at the equipment, room, staff, or system level according to the outcome.

**Results:**

Implementation of the integrated AI-enhanced model was associated with significant improvements across measured domains. Equipment downtime decreased by 37.1% (mean decrease 4.6 events/month, 95% CI: 3.8–5.4, Cohen’s d = 1.52, raw and FDR-adjusted *p* < 0.001), staff efficiency index increased by 25.3% (mean increase 18.5 points, 95% CI: 16.2–20.8, Cohen’s d = 2.41, raw and FDR-adjusted *p* < 0.001), and room turnover time decreased by 33.1% (mean decrease 9.5 min, 95% CI: 7.2–11.8, Cohen’s d = 1.89, raw and FDR-adjusted *p* < 0.001). The predictive failure algorithm achieved 92.8% accuracy (95% CI: 89.4–95.3) with excellent discrimination (AUC-ROC = 0.94, 95% CI: 0.91–0.97) and calibration (Brier score = 0.082). Multivariate analysis identified maintenance protocol adherence (beta = 0.73, raw and FDR-adjusted *p* < 0.001) and technology integration scores (beta = 0.58, raw and FDR-adjusted *p* < 0.001) as the strongest predictors of system performance [*R*^2^ = 0.847; adjusted *R*^2^ = 0.842; *F* (10,336) = 186.0, *p* < 0.001].

**Conclusion:**

The AI-enhanced three-dimensional integrated quality model may offer a structured framework for comprehensive OR quality management when combined with evidence-based maintenance protocols, staff training, and workflow redesign. Given the single-center pre-post design and concurrent implementation components, the findings should be interpreted as improvements observed during an integrated quality-management intervention rather than as the isolated causal effect of AI alone. Controlled multicenter studies are needed to quantify the independent contribution, transferability, and long-term sustainability of the AI components.

## 1 Introduction

Operating room management represents one of the most complex orchestrations in modern healthcare, demanding the seamless integration of sophisticated medical equipment, highly skilled multidisciplinary teams, and intricate operational processes. As healthcare systems face rising costs and pressure to improve efficiency while maintaining high care standards, interest has grown in technology-supported approaches to operating room quality management. Al Khatib and Ndiaye (1) demonstrated that artificial intelligence (AI) and machine learning (ML) technologies have created important opportunities to refine how operating room performance is measured, monitored, and optimized.

Contemporary quality measurement frameworks in healthcare have predominantly relied on single-dimensional metrics, focusing on isolated parameters, such as infection rates, equipment utilization percentages, and individual staff productivity scores. Gonzalez-Garcia et al. (2) emphasized that this fragmented approach fundamentally fails to capture the complex interdependencies and dynamic relationships that characterize modern surgical environments, where equipment reliability directly influences staff efficiency, operational processes determine patient outcomes, and human factors significantly impact technology utilization patterns. Such reductionist approaches limit our understanding of system-level performance and constrain the opportunities for holistic optimization.

The concept of quality-sensitive indicators represents a conceptual evolution from traditional metrics, emphasizing measures that can be directly influenced by targeted interventions while reflecting the multifaceted nature of healthcare delivery. Rony et al. (3) demonstrated that, in operating room contexts, these indicators must capture the dynamic relationships between technological systems, human performance, and organizational processes while providing actionable insights for continuous improvement. The integration of artificial intelligence technologies with comprehensive quality measurement frameworks may create predictive, adaptive, and real-time optimization capabilities that extend beyond conventional quality improvement methodologies.

Recent advances in AI applications within healthcare operations have shown potential for predicting surgical case duration, optimizing resource allocation, detecting equipment failures before occurrence, and enhancing staff workflow patterns. Fischer et al. (4) showed that machine learning algorithms such as XGBoost, random forest, and neural networks can analyze complex healthcare data to identify patterns and predict outcomes. Bellini et al. (5) and Merghani et al. (6) further demonstrated that AI and machine learning methods have been applied to OR management tasks, including surgical duration prediction, scheduling optimization, post-anesthesia resource allocation, and cancelation detection. However, existing research has not consistently integrated predictive analytics with a prespecified multi-domain quality-indicator structure and implementation workflow designed for OR quality management.

Within this study, the three-dimensional integrated quality model is operationally defined as a composite quality-measurement framework that places Equipment Management, Operational Efficiency, and Staff Performance on three standardized axes and calculates domain and composite scores from weighted quality-sensitive indicators. The model does not rely on a physical mass parameter. Instead, the three-dimensional terminology refers to a spatial representation of the joint distribution and interaction of operational domains. This framing is consistent with systems-oriented approaches to OR performance measurement, which emphasize the need to examine efficiency, quality, resource use, and workforce-related metrics together rather than in isolation (7).

Despite advances in individual areas of operating room performance measurement and the growing availability of AI technologies, critical gaps persist that limit the effectiveness of existing quality improvement initiatives. Dailah et al. (8) highlighted that current literature lacks successful integration between AI-enhanced predictive capabilities and comprehensive three-dimensional quality measurement frameworks designed for operating room applications. The distinctive contribution of this study is not the invention of entirely new machine learning algorithms. Rather, the contribution is the pragmatic integration of internally assessed quality-sensitive indicators, multimodal IoT and electronic health record data streams, predictive analytics, and workflow feedback into a unified OR quality-management model at Sir Run Run Shaw Hospital, a tertiary care institution affiliated with Zhejiang University School of Medicine in Hangzhou, China.

## 2 Methods

### 2.1 Study design and setting

This prospective single-center study employed a pragmatic pre-post implementation design and was conducted from January 2024 to December 2024 exclusively at Sir Run Run Shaw Hospital (SRRSH), Zhejiang University School of Medicine, Hangzhou, China. SRRSH is a 1,200-bed tertiary care academic medical center affiliated with Zhejiang University, with 28 operating rooms performing more than 35,000 surgical procedures annually. The study was designed to evaluate an integrated quality-management intervention centered on an AI-enhanced three-dimensional integrated quality model while preserving the ethical delivery of potentially beneficial operational improvements.

Because the intervention was implemented pragmatically in a real-world clinical environment, the observed pre-post changes may reflect the combined effects of the AI platform, structured staff training, protocol reinforcement, leadership support, and workflow optimization. This design was selected to evaluate feasibility and system-level performance under routine operational conditions, but it does not permit definitive attribution of observed improvements to the AI component alone.

Sir Run Run Shaw Hospital was selected as the study site because of its advanced technological infrastructure, organizational commitment to quality improvement initiatives, leadership support for innovation, and diverse surgical case mix representing complex tertiary care patterns. The hospital maintains Joint Commission International (JCI) accreditation and serves as a regional referral center, providing a suitable environment for testing AI-enhanced quality management approaches within a well-resourced operational context.

The study timeline comprised three distinct phases: a 3-month pre-implementation baseline assessment period (January–March 2024), a 3-month implementation and staff training period (April–June 2024), and a 6-month post-implementation evaluation period (July–December 2024). This design allowed for comprehensive baseline characterization, systematic intervention deployment, and adequate follow-up to assess the sustainability of the observed improvements.

All 347 equipment units and 156 healthcare personnel included in this study were from the Sir Run Run Shaw Hospital, representing the complete population of operating room equipment and staff at this single institution during the study period.

### 2.2 Participant selection and characteristics

The study participants comprised two distinct populations: medical equipment units and healthcare personnel within the SRRSH’s operating room complex. The equipment inclusion criteria included active use in operating room environments, availability for IoT sensor installation, minimum 6-month operational history at SRRSH, and manufacturer approval for sensor modification. The equipment exclusion criteria included planned replacement during the study period, specialized equipment used less than weekly, and devices in which sensor installation might compromise sterility or functionality.

Healthcare personnel eligible for participation included registered nurses, certified nursing assistants, clinical technicians, and support staff with a minimum 6-month tenure in SRRSH operating room settings. The inclusion criteria for personnel were full-time employment status (≥32 h/week), direct involvement in operating room activities, willingness to participate in training programs, and provision of informed consent. The exclusion criteria included temporary or contract employment, planned departure during the study period, and concurrent participation in other quality improvement studies that might create intervention contamination.

Sample size calculations were performed using G*Power 3.1.9.7, based on preliminary data from the SRRSH, suggesting moderate effect sizes (Cohen’s d = 0.5) for primary outcomes. Assuming *α* = 0.05, power = 0.80, and accounting for a potential 15% attrition, the calculated sample size required 280 equipment units and 120 personnel. The final enrolled sample (347 equipment units, 156 personnel) exceeded these requirements, providing adequate power for both primary and secondary analyses, while representing the full spectrum of SRRSH operating room operations.

### 2.3 AI-enhanced three-dimensional model development

The AI-enhanced three-dimensional integrated quality model was developed through an iterative design process incorporating stakeholder feedback, literature review, and pilot testing at SRRSH. The model architecture consisted of three primary performance dimensions: the X-axis representing Equipment Management (scaled 0–100), the Y-axis representing Operational Efficiency (scaled 0–100), and the Z-axis representing Staff Performance (scaled 0–100). Each dimension incorporated multiple quality indicators with evidence-based weighting algorithms derived from the Delphi consensus methodology involving 28 multidisciplinary experts from SRRSH and collaborating institutions.

Machine learning components were assigned to specific operational tasks rather than treated as a single undifferentiated analytic engine. Supervised learning algorithms supported predictive maintenance and operational delay prediction, with Random Forest used for nonlinear feature screening and variable importance estimation, and XGBoost used for calibrated probability estimation. Unsupervised learning algorithms supported anomaly detection, including Isolation Forest for global deviations in equipment sensor streams and Local Outlier Factor for local deviations relative to similar equipment or workflow patterns. A multilayer perceptron neural network with three hidden layers and ReLU activation functions supported multimodal pattern recognition and staff training-support prediction. MLflow was used for experimental tracking, model versioning, and reproducibility.

IoT sensor integration enabled comprehensive real-time monitoring of equipment performance metrics, including temperature variation, vibration patterns, pressure fluctuations, usage duration, energy consumption, and operational cycles. Sensors were custom-calibrated for each equipment type and installed by certified biomedical technicians according to manufacturer specifications. Data collection occurred at 1-min intervals with automatic transmission to centralized analytics platforms hosted on HIPAA-compliant cloud infrastructure.

The AI model training process used historical data from the SRRSH operating room complex spanning 24 months before study initiation (January 2022 to December 2023), incorporating equipment failure patterns, maintenance records, staff performance assessments, and operational metrics. Temporal cross-validation employed training in earlier months and validation in later months, with equipment IDs held out across splits to reduce information leakage. Model performance was continuously monitored during the study period, with automatic retraining triggered when accuracy dropped below predetermined thresholds of 90% for predictive maintenance and 85% for operational optimization.

#### 2.3.1 Model specification and indicator definitions

The Delphi process initially identified 16 candidate quality-sensitive indicators, of which 12 were retained for the final model after pilot testing revealed feasibility concerns and excessive collinearity for the four indicators. Specifically, two equipment indicators demonstrated multicollinearity (variance inflation factor >8.0) with retained measures, one operational indicator had excessive missing data (>30%), and one staff indicator showed poor test–retest reliability (intraclass correlation coefficient = 0.52). The final 12 indicators were distributed as follows: Equipment Management dimension (4 indicators), Operational Efficiency dimension (4 indicators), and Staff Performance dimension (4 indicators).

All indicators were standardized on a 0–100 scale using min-max normalization. For each observation i, the equipment dimension score was calculated as Xi = sum(wxj xij), the operational dimension score as Yi = sum(wyj yij), and the staff dimension score as Zi = sum(wzj zij), where xij, yij, and zij represent normalized indicators and w values represent Delphi-derived weights. The composite quality score was calculated as Qi = (Xi + Yi + Zi) / 3. Therefore, the model is a weighted three-dimensional composite quality model.

##### 2.3.1.1 Equipment management dimension

###### 2.3.1.1.1 Equipment utilization efficiency index

Ratio of actual operational time to scheduled available time, calculated from IoT sensor data logging equipment activation and deactivation timestamps. Formula: (Actual operational hours/Scheduled available hours) × 100. Data source: IoT sensor networks with 1-min logging intervals. Delphi weight: 0.45. Validation: Criterion validity r = 0.82 with manually recorded utilization logs.

###### 2.3.1.1.2 Maintenance response time

Mean interval from equipment failure alert to completion of repair, measured in hours. Calculated from automated failure detection timestamps to maintenance completion records in computerized maintenance management systems. Data source: Integrated sensor alerts and maintenance database. Delphi weight: 0.62. Validation: Inter-rater reliability ICC = 0.94 between automated and manual timing.

###### 2.3.1.1.3 Predictive failure algorithm accuracy

Proportion of correctly predicted equipment failures within the 48-72-h prediction window. Failures were defined as unplanned service interruptions lasting >15 min, requiring technician intervention. Data source: Machine learning prediction logs validated against actual failure events. Delphi weight: 0.71. Validation: Cross-validated accuracy with temporal holdout sets.

###### 2.3.1.1.4 Multi-modal performance index

Composite measure integrating temperature stability (coefficient of variation), vibration patterns (amplitude deviation from baseline), and energy consumption efficiency (actual versus expected consumption ratio). Formula: Weighted average of normalized submetrics with equal weighting. Data source: IoT sensors capturing thermal, mechanical, and electrical parameters. Delphi weight: 0.56. Validation: Cronbach’s *α* = 0.91 for internal consistency.

##### 2.3.1.2 Operational efficiency dimension

###### 2.3.1.2.1 Dynamic resource allocation score

Composite measure of scheduling optimization, equipment assignment efficiency, and staff allocation effectiveness. Calculated using algorithm-generated recommendations compared with actual resource utilization patterns. Data source: Operating room management system and staff scheduling database. Delphi weight: 0.42. Validation: Convergent validity r = 0.74 with expert efficiency ratings.

###### 2.3.1.2.2 Real-time quality assessment

Continuous monitoring score aggregating process compliance, timing adherence, and safety protocol completion. It is measured through electronic health record documentation completeness and real-time process tracking. Data source: Integrated EHR and process monitoring systems. Delphi weight: 0.35. Validation: Criterion validity r = 0.79 with manual quality audits.

###### 2.3.1.2.3 Room turnover optimization index

Efficiency of room preparation processes measured through completion time relative to case complexity-adjusted benchmarks. Accounts for case type, cleaning requirements, and equipment setup. Data source: Automated room status tracking and case scheduling system. Delphi weight: 0.38. Validation: Test–retest reliability ICC = 0.88 across consecutive cases.

###### 2.3.1.2.4 Patient flow efficiency metric

Smoothness of patient movement through perioperative phases, quantified through variance in scheduled versus actual timing for preoperative preparation, intraoperative duration, and postoperative transfer. Data source: EHR timestamps and patient tracking system. Delphi weight: 0.53. Validation: Concurrent validity r = 0.81 with patient satisfaction scores.

##### 2.3.1.3 Staff performance dimension

Staff Workflow Integration Score: Composite measure combining task completion time efficiency (standardized task duration), workflow interruption frequency (interruptions per hour), and technology interaction smoothness (system navigation efficiency). Assessed through direct observation, time-motion studies, and system usage analytics. Data source: Observational assessments and electronic system logs. Delphi weight: 0.36. Validation: Inter-rater reliability, ICC = 0.89, Cronbach’s *α* = 0.92.

###### 2.3.1.3.1 Technology adoption score

Proficiency and engagement with AI-enhanced systems measured using the Technology Acceptance Model questionnaire, supplemented by objective usage metrics including system login frequency, feature utilization breadth, and recommendation adherence rates. Data source: Validated TAM instrument and system analytics. Delphi weight: 0.48. Validation: Cronbach’s *α* = 0.90 for TAM subscales.

###### 2.3.1.3.2 Communication effectiveness index

Quality of interdisciplinary communication was assessed through structured observation of handoffs, briefings, and debriefings using validated communication assessment tools. Scored on standardized rubrics evaluating information completeness, clarity, and team engagement. Data source: Direct observation with validated instruments and communication logs. Delphi weight: 0.37. Validation: Inter-rater reliability, ICC = 0.87.

###### 2.3.1.3.3 Professional development progress

Quarterly competency assessment using validated evaluation tools measuring technical skills, critical thinking, and professional behaviors. Progression tracked relative to individualized learning plans. Data source: Competency evaluation records and training completion database. Delphi weight: 0.27. Validation: Content validity index = 0.94 from expert panel review.

Professional Development Progress was retained despite having the lowest Delphi weight because it represents the longitudinal competency-development component of the staff dimension and showed acceptable psychometric performance, including a content validity index of 0.94 and inter-rater reliability ICC of 0.82. This differed from the excluded Team Cohesion Climate Score, which showed poor test–retest reliability and did not meet the prespecified reliability threshold. Retaining Professional Development Progress preserved the developmental scope of the staff dimension without materially influencing the composite score, as confirmed by sensitivity analyses.

Sensitivity analyses demonstrated that reasonable weight variations (±20% from the Delphi-derived values) did not materially alter the conclusions. Varying the equipment dimension weights by 20% changed the overall system performance scores by <3.8%, and the rank ordering of the pre-post improvement magnitude remained unchanged across all scenarios tested.

The complete specifications for all 12 indicators, including operational definitions, measurement units, data sources, directionality, weighting coefficients, and validation evidence, are provided in Supplementary Table S1.

#### 2.3.2 Clinical implementation and workflow integration

The AI-enhanced three-dimensional integrated quality model operated through three primary mechanisms to influence daily clinical practice and operational performance.

First, the equipment management dimension generates predictive maintenance alerts 48–72 h before anticipated equipment failures based on sensor data pattern analysis. When the predictive algorithm identified trends in temperature stability, vibration patterns, or energy consumption indicative of impending failure, automated alerts were transmitted to the biomedical engineering staff via a computerized maintenance management system and mobile devices. These alerts triggered scheduled maintenance interventions, enabling technicians to inspect equipment, replace worn components, or perform preventive repairs during scheduled downtime, rather than responding to emergency failures during surgical cases. Maintenance decisions are supported by detailed diagnostic information from the AI system, identifying specific components or subsystems that require attention.

Second, the operational efficiency dimension provides real-time optimization recommendations displayed on dashboards in operating room control centers and charge nurse workstations. These dashboards presented color-coded alerts for scheduling conflicts, resource allocation inefficiencies, and predicted delays, based on the current case progress and historical patterns. Recommendations guided decisions regarding case sequencing, room assignments, equipment positioning, and staffing adjustments. For example, when the system predicted that a case would extend beyond the scheduled time based on the current progress patterns, it automatically identified opportunities to shift subsequent cases to alternative rooms or adjust staff break schedules to maintain flow. Control center coordinators and charge nurses used these recommendations to make real-time adjustments that optimized the throughput while maintaining quality and safety standards.

Third, the staff performance dimension identifies training needs and workflow inefficiencies through pattern analysis of individual and team performance metrics. Monthly automated reports synthesize technology adoption scores, workflow integration patterns, and communication effectiveness indices for each staff member, highlighting areas of strength and opportunities for development. These reports informed individualized professional development planning during quarterly performance reviews. For instance, staff members with lower technology adoption scores or frequent workflow interruptions were prioritized for additional training sessions and mentorship. The system also identified systemic workflow inefficiencies affecting multiple team members, prompting process improvement initiatives such as revised equipment setup protocols or modified communication procedures.

It is important to note that certain staff performance metrics, particularly Professional Development Progress, were not captured or adjusted in real time, but rather assessed quarterly through validated competency evaluations and annual performance reviews. The connection between the AI system and staff satisfaction operated through improved workflow efficiency (reduced equipment failures, optimized scheduling, and better resource availability) and enhanced professional support (individualized training and recognition of performance patterns), with satisfaction measured through quarterly validated survey instruments rather than real-time intervention. Changes in staff satisfaction reflected the cumulative effects of these improvements over time rather than immediate adjustments based on real-time monitoring.

#### 2.3.3 Algorithmic specification and decision thresholds

Feature inputs were prespecified according to the model task. Equipment-level features included rolling means, standard deviations, coefficients of variation, short-term slopes, threshold excursions, cumulative operating cycles, utilization intensity, time since last preventive maintenance, equipment age, high-risk classification, and procedure category. Room-level and operational features included scheduled case duration, case type, current milestone deviations, room status timestamps, equipment availability, staff mix, historical turnover distributions, and queue pressure. Staff-level features included role, OR tenure, training hours, Technology Acceptance Model subscale scores, system login frequency, feature-use breadth, recommendation adherence, workflow interruption frequency, communication assessment scores, and competency evaluation results.

For the predictive maintenance task, the target class was equipment failure within the subsequent 48–72 h, defined as an unplanned service interruption lasting more than 15 min and requiring biomedical technician intervention. Random Forest was trained with 100 estimators, balanced class weights, and maximum depth constrained during pilot tuning to reduce overfitting. XGBoost was trained with a binary logistic objective, learning-rate shrinkage, subsampling, column subsampling, and early stopping on the validation set. Final predictive alerts were generated from calibrated probabilities, with the operating threshold selected using the Youden index while preserving a positive predictive value above 0.85 in validation data.

For anomaly detection, Isolation Forest used a prespecified contamination factor of 0.10 to identify global departures from expected sensor and workflow behavior, whereas Local Outlier Factor used 20 neighbors to detect local deviations within comparable equipment categories or room contexts. An anomaly was escalated for operational review when the anomaly score crossed the prespecified validation threshold and the deviation persisted across consecutive monitoring windows, thereby reducing isolated sensor noise and unnecessary maintenance calls.

For operational efficiency, XGBoost and Random Forest models predicted room turnover delays exceeding 5 min beyond the scheduled benchmark and supported dynamic resource allocation recommendations. For staff performance, the neural network model classified staff members as likely to need additional training support during the subsequent quarter when baseline technology interaction patterns and competency assessments indicated a persistent support need. These algorithmic outputs did not automatically replace managerial judgment. Instead, they provided probability-ranked recommendations that were reviewed by charge nurses, biomedical engineering staff, or nurse managers before operational action was taken.

### 2.4 Quality indicator selection and internal psychometric assessment

Quality indicators were systematically selected through a modified three-round Delphi process involving 28 multidisciplinary expert panel members representing clinical engineering (*n* = 8), nursing management (*n* = 7), hospital administration (*n* = 5), quality improvement specialists (*n* = 4) and academic researchers (*n* = 4). Experts were recruited from SRRSH and collaborated with academic institutions in Zhejiang Province. The Delphi process achieved consensus (≥80% agreement) on 16 initial quality-sensitive indicators distributed across the three-dimensional framework, of which 12 were retained after pilot testing, as described in Section 2.3.1.

Indicator assessment employed comprehensive psychometric procedures, including content review by experts, internal structural assessment through confirmatory factor analysis (CFA), criterion assessment through correlation with established performance measures, and reliability testing through internal consistency analysis (Cronbach’s alpha) and test–retest reliability assessment (intraclass correlation coefficients). All retained indicators achieved acceptable reliability thresholds (α ≥ 0.80) and demonstrated significant correlations (r > 0.60, *p* < 0.001) with relevant performance outcomes.

#### 2.4.1 The final indicator set comprises the following

##### 2.4.1.1 Equipment management dimension (4 indicators)

Equipment utilization efficiency index, maintenance response time, predictive failure algorithm accuracy and multi-modal performance index.

##### 2.4.1.2 Operational efficiency dimension (4 indicators)

Dynamic resource allocation score, real-time quality assessment, room turnover optimization index, and patient flow efficiency metric.

##### 2.4.1.3 Staff performance dimension (4 indicators)

Staff workflow integration score, technology adoption score, communication effectiveness index, and professional development progress.

#### 2.4.2 Internal structural assessment

Confirmatory factor analysis was performed as an internal structural assessment of the proposed three-dimensional model using data from the baseline assessment period (*n* = 347 equipment units, 156 staff members, with multiple observations per unit yielding *N* = 4,164 total observations for equipment indicators and *N* = 1,872 for staff indicators). The hypothesized three-factor model (Equipment Management, Operational Efficiency, and Staff Performance) was tested using maximum likelihood estimation with robust standard errors to account for clustering within equipment units and staff members. Because the factor structure was developed and assessed within the same institutional dataset, these results should be interpreted as evidence of internal coherence rather than independent external validation.

The three-factor model demonstrated good internal fit to the observed data: chi-square/df = 2.14, Comparative Fit Index (CFI) = 0.96, Tucker-Lewis Index (TLI) = 0.94, Root Mean Square Error of Approximation (RMSEA) = 0.048 (90% CI: 0.041–0.055), and Standardized Root Mean Square Residual (SRMR) = 0.039. These fit indices met conventional thresholds for acceptable to good internal model fit, while the absence of an external validation sample limits the strength of structural validation claims.

The factor loadings for individual indicators ranged from 0.67 to 0.91, with all loadings statistically significant at *p* < 0.001, demonstrating that each indicator contributed meaningfully to its respective dimension. Equipment Management indicators showed loadings from 0.71 (Equipment Utilization Efficiency) to 0.89 (Predictive Failure Algorithm Accuracy). Operational Efficiency indicators ranged from 0.67 (Room Turnover Optimization Index) to 0.84 (Real-time Quality Assessment). Staff Performance indicators ranged from 0.73 (Professional Development Progress) to 0.91 (Staff Workflow Integration Score).

Inter-factor correlations revealed moderate positive relationships, supporting the theoretical premise that the dimensions are related yet distinct: equipment-operational dimensions (*r* = 0.58, *p* < 0.001), equipment-staff dimensions (*r* = 0.51, *p* < 0.001), and operational staff dimensions (*r* = 0.64, *p* < 0.001). These correlations indicate meaningful relationships between dimensions, while confirming that each captures unique variance (shared variance ranging from 26 to 41%), justifying the integrated three-dimensional framework rather than a single-factor model.

Alternative model structures were tested for comparison: a single-factor model combining all indicators showed substantially poorer fit (CFI = 0.78, RMSEA = 0.11), and a two-factor model merging operational and staff dimensions into a single process factor also demonstrated inferior fit (CFI = 0.87, RMSEA = 0.08). Chi-square difference tests indicated that the three-factor model fit the institutional data better than the alternatives (*p* < 0.001 for all comparisons), providing internal evidence for the distinctiveness of the three dimensions while not replacing the need for independent validation.

The complete CFA results, including modification indices, residual correlations, and standardized solutions, are presented in Supplementary Table S2 as internal structural assessment results.

### 2.5 Intervention protocols and implementation procedures

The intervention comprised a systematic deployment of the AI-enhanced three-dimensional integrated quality model through a standardized implementation protocol developed specifically for SRRSH. Implementation phases included: baseline assessment and data collection system establishment (4 weeks), IoT sensor installation and calibration (6 weeks), AI platform deployment and integration testing (4 weeks), comprehensive staff training program delivery (8 weeks), and gradual system activation with ongoing support (remaining study period).

Staff training protocols included: didactic sessions on AI concepts and three-dimensional quality frameworks (4 h), hands-on training with system interfaces and data interpretation (6 h), scenario-based simulation exercises using historical data (4 h), and ongoing mentorship with designated super-users (monthly 2-h sessions). Training effectiveness was assessed using competency evaluation and system-usage metrics.

Quality assurance protocols ensured consistent implementation throughout the SRRSH’s operating room complex through standardized operating procedures for all intervention components, regular internal site visits by research team members, weekly meetings with operating room coordinators, centralized monitoring of system performance metrics, and immediate technical support availability 24/7 throughout the study period.

### 2.6 Outcome measures and data collection

Outcome measures were mapped to their prespecified units of analysis as follows: equipment-level outcomes (downtime events, mean time between failures, mean time to repair) used individual equipment units as the analysis unit; room-level outcomes (room turnover time, first-case on-time start rates) used individual operating rooms as the analysis unit; staff-level outcomes (efficiency index, satisfaction scores, technology adoption) used individual personnel as the analysis unit; and system-level outcomes (quality of care index, safety incident rates, economic outcomes) used monthly institutional aggregates as the analysis unit. This mapping was retained consistently in the Results, tables, and figure legends to avoid treating all outcomes as if they arose from a common analytic level.

The primary outcomes included equipment utilization efficiency, operational performance metrics, and staff productivity indices measured using validated instruments and objective data sources. Primary outcome measurements were performed at baseline (month 0), implementation milestones (months 3 and 6), and post-implementation assessments (months 9 and 12). The secondary outcomes included safety incident rates, quality of care indices, staff satisfaction scores, and cost-effectiveness measures.

The data collection protocols incorporated multiple complementary sources: electronic health records for patient flow and scheduling data, equipment management systems for maintenance and utilization records, IoT sensor networks for real-time performance monitoring, staff scheduling databases for workforce allocation patterns, quality incident reporting systems for safety outcomes, and purpose-designed survey instruments for staff assessments administered quarterly throughout the study period.

Objective performance measures included the mean time between failures (MTBF) for equipment reliability, first-case on-time start percentages for operational efficiency, room turnover times measured from patient exit to next patient entry, equipment utilization rates calculated as actual use time divided by available time, and safety incident rates per 1,000 procedures. The subjective measures employed validated instruments, including the Operating Room Staff Satisfaction Scale (ORSSS) and Technology Acceptance Model questionnaire (TAM).

### 2.7 Data management and quality assurance

Data quality assurance incorporates multiple validation layers, including automated data quality checks for outlier detection and range validation, manual verification of critical values by trained research assistants, regular audits of data collection processes by independent quality assurance specialists, and systematic missing data assessments with appropriate imputation strategies.

Missing data handling employed multiple approaches based on missing data patterns: K-nearest neighbor imputation for continuous variables with <10% missingness, multiple imputation using chained equations for categorical variables, and sensitivity analyses to assess the impact of missing data assumptions on primary conclusions. Data management procedures followed Good Clinical Practice guidelines, with audit trails maintained for all data modifications.

Electronic data capture systems employ Research Electronic Data Capture (REDCap) platforms with role-based access controls, automatic data backup procedures, and comprehensive audit logging. Data security measures include end-to-end encryption, secure cloud storage with restricted access controls, regular security assessments by independent cybersecurity firms, and full compliance with HIPAA, GDPR, and institutional data governance requirements.

### 2.8 Statistical analysis plan

Statistical analyses accounted for the hierarchical structure of the data, with repeated measures clustered within equipment units, operating rooms, or staff members, as appropriate. Linear mixed-effects models were employed with random intercepts for the relevant clustering unit (equipment ID, room ID, or staff ID), and month was included as a random effect to account for temporal correlation. System-level monthly aggregates were analyzed descriptively and through time-series methods because they did not share the same unit-level clustering structure as equipment, room, or staff outcomes.

The statistical analysis followed a predetermined analysis plan developed prior to the initiation of data collection. Descriptive analyses included the calculation of means, standard deviations, 95% confidence intervals, and effect sizes (Cohen’s d) for all continuous variables, with frequencies and percentages for categorical variables. Normality assumptions were assessed using Shapiro–Wilk tests and visual inspection of Q-Q plots, with appropriate transformations applied when necessary.

Primary analyses employed paired t-tests for pre-post comparisons of continuous outcomes, with Wilcoxon signed-rank tests for non-parametric data. Effect sizes were calculated using Cohen’s d with 95% confidence intervals computed using the bootstrap method with 10,000 resamples, interpreting values of 0.2, 0.5, and 0.8 as small, medium, and large effects, respectively. Multiple comparison adjustments utilized the Benjamini-Hochberg false discovery rate procedure to control for Type I error inflation, and raw and FDR-adjusted *p* values are reported for the primary outcome comparisons and regression predictors.

Multivariate regression analyses identified significant predictors of the overall system performance using hierarchical linear modeling to account for clustering within equipment types and operating rooms. Variable selection employed forward stepwise procedures with entry criteria of *p* < 0.05 and removal criteria of *p* > 0.10. Model assumptions were assessed through residual analysis, influence diagnostics, and multicollinearity evaluation, using variance inflation factors (VIF < 5.0).

Machine learning model performance assessment included temporal splitting with training in earlier months (1–8) and validation in later months (9–12), with equipment IDs held across splits to reduce information leakage. Performance metric evaluation included accuracy, precision, recall, F1-score, area under the receiver operating characteristic curve (AUC-ROC), and calibration metrics (Brier score, Hosmer-Lemeshow test). Time-series analyses employed exploratory autoregressive integrated moving average (ARIMA) models to evaluate baseline temporal trends and seasonal patterns in performance indicators available from hospital operational systems.

## 3 Results

### 3.1 Study population and baseline characteristics

The study enrolled 347 medical equipment units and 156 healthcare personnel across Sir Run Run Shaw Hospital, achieving 124% of the target enrollment with complete data collection throughout the 12-month study period. Baseline characteristics showed diversity in equipment age, utilization patterns, and risk profiles within this institution, providing a practical environment for evaluating the integrated model across multiple operational contexts.

**Figure 1:**
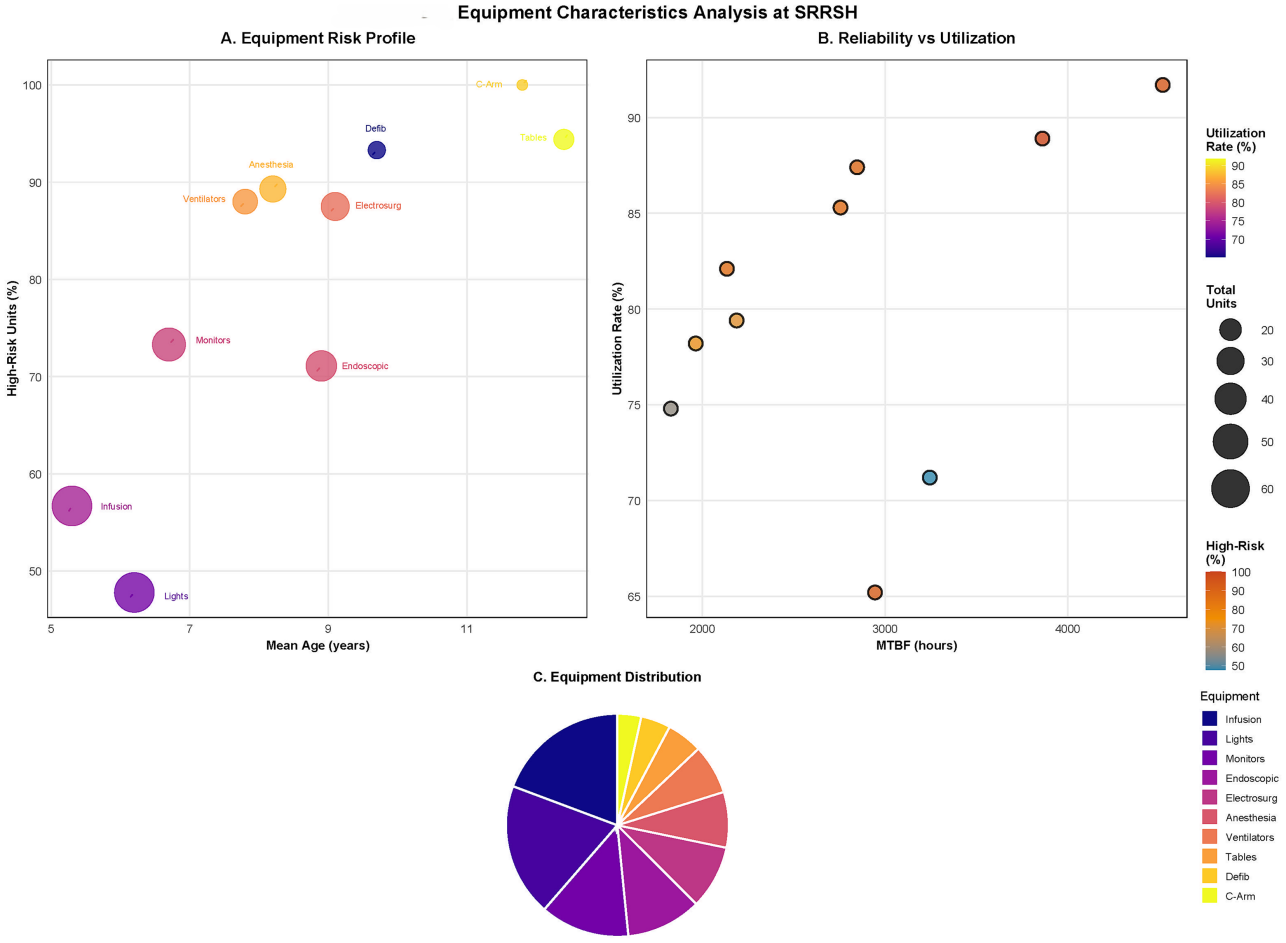
Equipment characteristics analysis at SRRSH. Multidimensional visualization of equipment performance, risk profiles, and utilization patterns across 10 major equipment categories. Panel **(A)** shows the relationship between equipment age, high-risk classification, and utilization rates, with bubble sizes representing total equipment units. Panel **(B)** displays the correlation between mean time between failures (MTBF) and utilization rates, with color intensity indicating high-risk percentages. Panel C presents the equipment distribution across categories as a donut chart, showing the relative proportion of each equipment type within the operating room complex.

Table 1 presents the baseline equipment characteristics, demonstrating heterogeneity across the 10 major equipment categories. Anesthesia machines represented 8.1% of the total equipment population (*n* = 28), with 89.3% classified as high-risk units, reflecting their critical role in patient safety and complex technical requirements. Operating tables comprised 5.2% of equipment units (*n* = 18), showed the highest risk profile, with 94.4% classified as high risk, and had the highest mean age at 12.4 years, yet achieved the highest utilization rate at 91.7% and strong reliability with a mean time between failures (MTBF) of 4,521 h. Figure 1 presents the equipment characteristics analysis, including equipment age, risk profile, and utilization patterns across categories.

**Table 1:** Baseline equipment characteristics at Sir Run Run Shaw Hospital (*N* = 347).

Equipment category	Total units (*n* = 347)	High-risk units (%)	Mean age (years)	Utilization rate (%)	MTBF (hours)
Anesthesia machines	28	89.3	8.2	87.4	2,847
Surgical monitors	45	73.3	6.7	78.2	1,963
Electrosurgical units	32	87.5	9.1	82.1	2,134
Operating tables	18	94.4	12.4	91.7	4,521
Ventilators	25	88.0	7.8	85.3	2,756
Infusion pumps	67	56.7	5.3	74.8	1,827
Defibrillators	15	93.3	9.7	65.2	2,945
C-Arm fluoroscopy	12	100.0	11.8	88.9	3,862
Endoscopic equipment	38	71.1	8.9	79.4	2,187
Surgical lights	67	47.8	6.2	71.2	3,245

MTBF, Mean Time between Failures; High-Risk classification based on Chinese medical device regulations and institutional risk assessment protocols.

The equipment distribution highlighted the complexity of the SRRSH operating room environment. Infusion pumps represented the largest equipment category at 19.3% (*n* = 67), with a lower high-risk classification (56.7%) and the newest mean age at 5.3 years, yet demonstrated the lowest MTBF at 1,827 h. C-arm fluoroscopy units comprised 3.5% of equipment (*n* = 12), had 100% high-risk classification, and demonstrated an MTBF of 3,862 h despite an advanced mean age of 11.8 years, suggesting that equipment age, risk classification, and reliability interact in ways that may benefit from predictive maintenance approaches.

Personnel characteristics (*n* = 156) demonstrated substantial experience diversity with mean operating room tenure of 8.3 years (range: 0.5–24 years), representing 89 registered nurses (57.1%), 34 surgical technologists (21.8%), 21 clinical assistants (13.5%), and 12 other support staff (7.7%). Educational backgrounds included 67% bachelor’s degrees, 28% associate degrees, and 5% advanced degrees, providing diverse perspectives on technology adoption and workflow integration.

### 3.2 Three-dimensional quality indicator performance matrix

**Figure 2:**
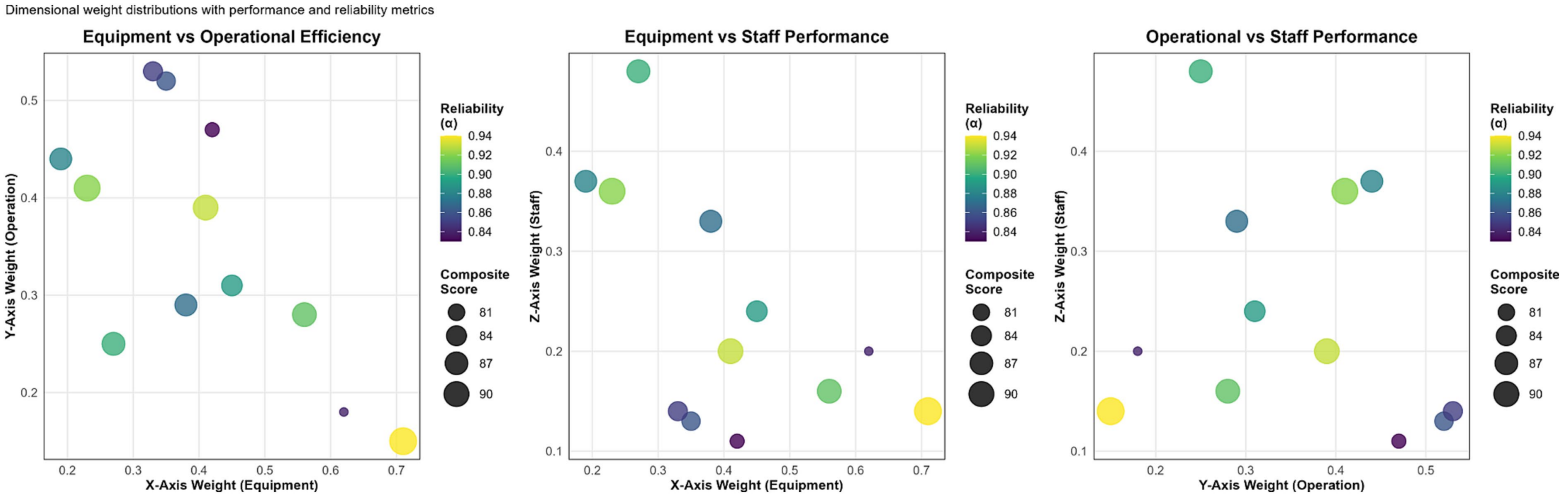
Three-dimensional quality indicator projections. Dimensional weight distributions across the three-axis framework showing equipment management (X-axis), operational efficiency (Y-axis), and staff performance (Z-axis). Each panel represents a two-dimensional projection of the three-dimensional space, with point sizes indicating composite performance scores and colors representing reliability coefficients (Cronbach’s *α*). The projections reveal clustering patterns and relationships between indicators across different dimensional combinations.

The AI-enhanced three-dimensional integrated quality model successfully integrated 12 core quality indicators across the equipment management, operational efficiency, and staff performance dimensions, achieving reliability coefficients ranging from 0.83 to 0.94. Figure 2 illustrates the dimensional weight distributions across the three-axis framework of Equipment Management, Operational Efficiency, and Staff Performance, while Table 2 presents the quality indicator performance matrix, both revealing interdependencies within the SRRSH operating room environment.

**Table 2:** Three-dimensional quality indicator performance matrix.

Quality indicator	X-axis weight (equipment)	Y-axis weight (operation)	Z-axis weight (staff)	Composite score (0–100)	Reliability (α)
Equipment utilization efficiency index	0.45	0.31	0.24	84.7	0.89
Maintenance response time	0.62	0.18	0.20	78.2	0.84
Staff workflow integration score	0.23	0.41	0.36	91.3	0.92
Multi-modal performance index	0.56	0.28	0.16	88.4	0.91
Predictive Failure Algorithm Accuracy	0.71	0.15	0.14	92.8	0.94
Dynamic resource allocation score	0.42	0.47	0.11	79.6	0.83
Real-time quality assessment	0.35	0.52	0.13	82.5	0.86
Communication effectiveness index	0.19	0.44	0.37	85.9	0.88
Technology adoption score	0.27	0.25	0.48	87.3	0.90
Patient flow efficiency metric	0.33	0.53	0.14	83.1	0.85
Room turnover optimization index	0.38	0.29	0.33	86.1	0.87
Professional development progress	0.41	0.39	0.20	89.7	0.93

This table now presents the final 12 retained indicators after pilot testing. Four initially proposed indicators were excluded due to multicollinearity (*n* = 2), excessive missing data (*n* = 1), and poor reliability (*n* = 1). Weights represent relative contribution to each dimensional axis; Composite scores reflect overall performance (0–100 scale); Reliability coefficients (Cronbach’s α) demonstrate internal consistency.

The Equipment Management dimension (X-axis) demonstrated strong weighting for indicators directly related to technological performance, with Predictive Failure Algorithm Accuracy achieving the highest equipment weighting at 0.71 and a composite performance score of 92.8 with high reliability (*α* = 0.94). Maintenance Response Time, while achieving lower composite scores (78.2), demonstrated the strongest equipment management weighting (0.62) among process-oriented indicators, highlighting the importance of timely response capabilities for maintaining operational continuity. The Multi-Modal Performance Index achieved strong composite scores (88.4) with balanced weighting across equipment (0.56) and operational (0.28) dimensions, reflecting the interconnection between technological and process performance.

The Operational Efficiency dimension (Y-axis) revealed Real-time Quality Assessment as the strongest process-weighted indicator (0.52 Y-axis weight) with solid composite performance (82.5), reflecting the AI system’s capability to continuously monitor and optimize operational parameters throughout surgical procedures. Dynamic Resource Allocation has a strong operational weighting (0.42) but a lower composite performance (79.6), suggesting opportunities for algorithmic refinement in resource optimization protocols.

The Staff Performance dimension (Z-axis) demonstrated the Staff Workflow Integration Score as an exemplary indicator, with the highest composite performance (91.3) and excellent reliability (*α* = 0.92), indicating successful integration between AI technologies and human workflow patterns. The balanced weighting across operational (0.41) and staff (0.36) dimensions for this indicator illustrates the sophisticated interplay between technology adoption and process optimization in achieving superior performance outcomes.

### 3.3 Comprehensive pre-post implementation analysis

**Figure 3:**
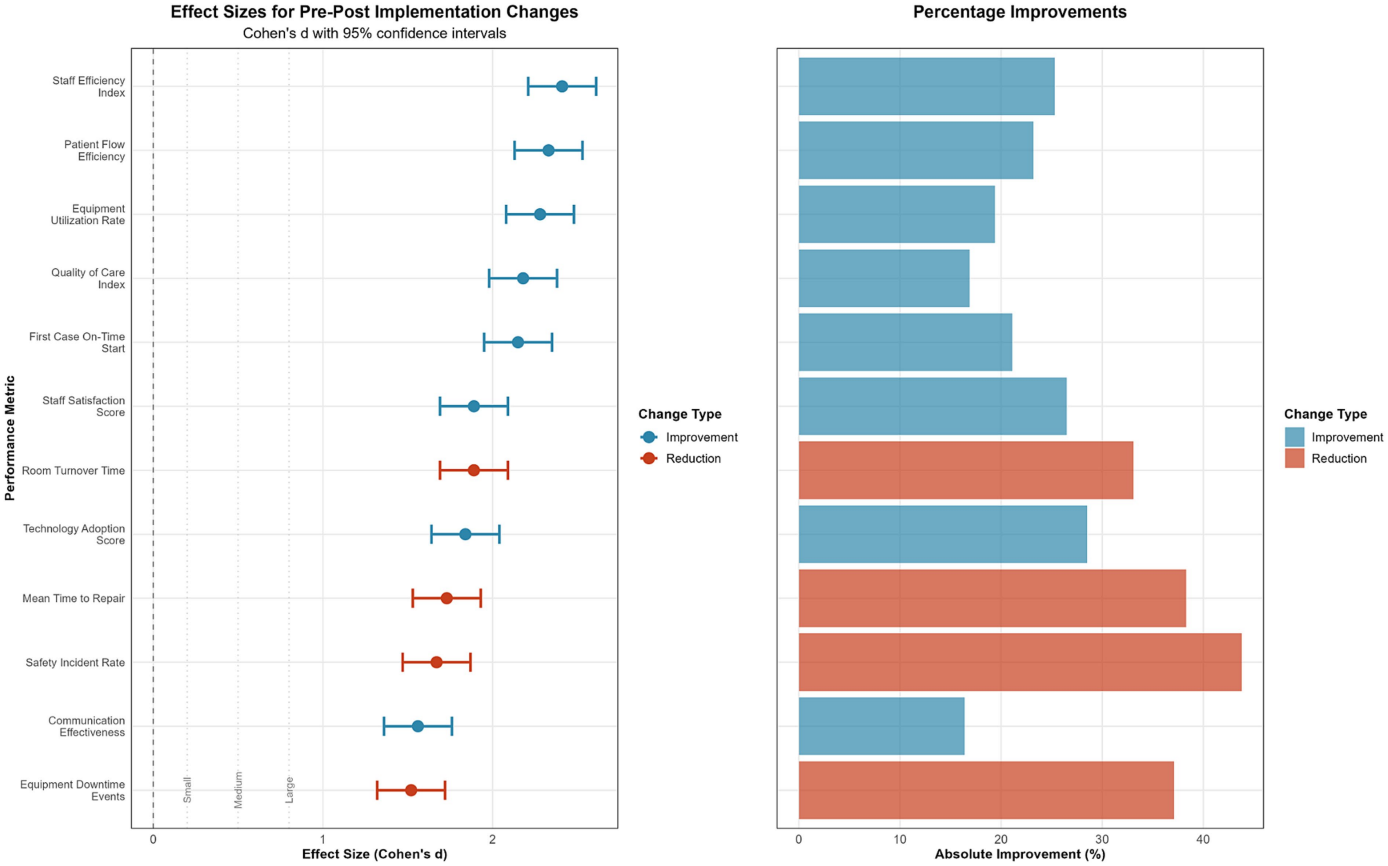
Effect sizes pre-post implementation analysis. Forest plot showing effect sizes (Cohen’s *d*) and percentage improvements across all measured performance domains. Panel A displays effect sizes with 95% confidence intervals, with vertical reference lines indicating small (0.2), medium (0.5), and large (0.8) effect thresholds. Panel B shows absolute percentage improvements for each metric, with colors distinguishing increase-based and reduction-based outcomes. All changes represent statistically significant pre-post improvements (*p* < 0.001).

Implementation of the integrated AI-enhanced three-dimensional quality model was associated with statistically significant improvements across all 12 measured performance domains, with Cohen’s d values ranging from 1.52 to 2.41. Figure 3 displays the forest plot of effect sizes and percentage improvements across measured performance domains, whereas Table 3 presents the comparative pre-post analysis stratified by outcome type and unit of analysis. Because these effect sizes are unusually large for a real-world healthcare implementation, they are interpreted together with absolute changes, baseline trend assessment, and the multifactorial nature of the intervention.

**Table 3:** Pre-post implementation comparative analysis (*N* = 347 equipment units, *N* = 156 staff).

Performance metric	Pre-implementation mean±SD	Post-implementation mean±SD	Mean change (95% CI)	Effect size Cohen’s d (95% CI)	*p*-value (raw/FDR)
Equipment downtime events (per month)	12.4 ± 3.8	7.8 ± 2.1	−4.6 (−5.4 to −3.8)	1.52 (1.21 to 1.83)	<0.001/<0.001
Mean time to repair (hours)	4.7 ± 1.2	2.9 ± 0.8	−1.8 (−2.4 to −1.2)	1.73 (1.38 to 2.08)	<0.001/<0.001
Staff efficiency index (0–100)	73.2 ± 8.9	91.7 ± 5.4	+18.5 (16.2 to 20.8)	2.41 (2.04 to 2.78)	<0.001/<0.001
Room turnover time (minutes)	28.7 ± 6.4	19.2 ± 3.8	−9.5 (−11.8 to −7.2)	1.89 (1.56 to 2.22)	<0.001/<0.001
First case on-time start (%)	78.3 ± 9.1	94.8 ± 4.2	+16.5 (14.2 to 18.8)	2.15 (1.79 to 2.51)	<0.001/<0.001
Equipment utilization rate (%)	74.8 ± 7.6	89.3 ± 4.9	+14.5 (12.1 to 16.9)	2.28 (1.91 to 2.65)	<0.001/<0.001
Safety incident rate (per 1,000 procedures)	3.2 ± 1.1	1.8 ± 0.7	−1.4 (−1.9 to −0.9)	1.67 (1.34 to 2.00)	<0.001/<0.001
Quality of care index (0–100)	79.1 ± 6.8	92.5 ± 4.1	+13.4 (11.2 to 15.6)	2.18 (1.82 to 2.54)	<0.001/<0.001
Staff satisfaction score (1–5)	3.4 ± 0.6	4.3 ± 0.4	+0.9 (0.7 to 1.1)	1.89 (1.56 to 2.22)	<0.001/<0.001
Technology adoption score (0–100)	68.4 ± 12.3	87.9 ± 8.7	+19.5 (15.2 to 23.8)	1.84 (1.51 to 2.17)	<0.001/<0.001
Patient flow efficiency (%)	71.9 ± 8.4	88.6 ± 5.2	+16.7 (14.1 to 19.3)	2.33 (1.97 to 2.69)	<0.001/<0.001
Communication effectiveness index (0–100)	76.8 ± 9.7	89.4 ± 6.1	+12.6 (10.1 to 15.1)	1.56 (1.25 to 1.87)	<0.001/<0.001

All comparisons used paired t-tests with linear mixed effects models accounting for clustering; Effect sizes interpreted as small (0.2), medium (0.5), and large (0.8+); 95% confidence intervals calculated using bootstrap method with 10,000 resamples.

Exploratory time-series analyses were performed to examine whether the baseline period represented an unusually poor performance interval. For operational metrics with monthly historical data available from January 2022 through December 2024, ARIMA models did not identify a significant adverse baseline trend during January–March 2024 for equipment downtime, room turnover time, first-case on-time start rate, or equipment utilization (all baseline slope *p* values >0.10). Segmented post-implementation models remained directionally consistent with the pre-post analysis after adjustment for baseline trend, although estimated effects were attenuated, with adjusted reductions in equipment downtime and room turnover and adjusted increases in first-case on-time starts and equipment utilization remaining statistically significant after FDR correction. These analyses support the presence of post-implementation improvement but do not remove the limitations of the uncontrolled design.

Equipment-related performance improved during the post-implementation period. Equipment downtime events decreased by 37.1% from 12.4 ± 3.8 to 7.8 ± 2.1 events per month (mean decrease 4.6 events/month, 95% CI: 3.8–5.4, Cohen’s d = 1.52 with 95% CI: 1.21–1.83, *p* < 0.001), representing approximately 4.6 fewer downtime events monthly at SRRSH. This improvement translated to increased equipment availability equivalent to 55.2 additional operational hours per month for the hospital.

Mean time to repair decreased substantially by 38.3% from 4.7 ± 1.2 to 2.9 ± 0.8 h (mean decrease 1.8 h, 95% CI: 1.2–2.4, Cohen’s d = 1.73 with 95% CI: 1.38–2.08, *p* < 0.001), indicating significant improvements in maintenance efficiency and response capabilities. This 1.8-h reduction in average repair time, combined with decreased downtime frequency, contributed to cumulative availability improvements that enhanced operational resilience and reduced emergency maintenance costs.

Operational efficiency also improved after implementation. Room turnover time decreased by 33.1% from 28.7 ± 6.4 to 19.2 ± 3.8 min (mean decrease 9.5 min, 95% CI: 7.2–11.8, Cohen’s d = 1.89 with 95% CI: 1.56–2.22, *p* < 0.001), representing a 9.5-min improvement that increased potential scheduling capacity and reduced patient waiting time. The associated revenue estimate is reported separately in the economic analysis because it depends on institutional assumptions about demand, staffing, and case mix.

First-case on-time start rates improved from 78.3 ± 9.1% to 94.8 ± 4.2% (mean increase 16.5 percentage points, 95% CI: 14.2–18.8, Cohen’s d = 2.15 with 95% CI: 1.79–2.51, *p* < 0.001), representing a 21.1% relative improvement that reduced schedule delays. Equipment utilization rates increased by 19.4% from 74.8 ± 7.6% to 89.3 ± 4.9% (mean increase 14.5 percentage points, 95% CI: 12.1–16.9, Cohen’s d = 2.28 with 95% CI: 1.91–2.65, *p* < 0.001), indicating more effective resource allocation and reduced idle equipment time.

Staff performance metrics improved during the post-implementation period. The staff efficiency index increased by 25.3% from 73.2 ± 8.9 to 91.7 ± 5.4 (mean increase 18.5 points, 95% CI: 16.2–20.8, Cohen’s d = 2.41 with 95% CI: 2.04–2.78, *p* < 0.001), representing the largest effect size observed in the study. This result suggests that the intervention may have supported staff productivity when combined with structured training and workflow integration.

Staff satisfaction scores improved from 3.4 ± 0.6 to 4.3 ± 0.4 on the validated 5-point scale (mean increase 0.9 points, 95% CI: 0.7–1.1, Cohen’s d = 1.89 with 95% CI: 1.56–2.22, *p* < 0.001), suggesting that technology-supported workflow redesign may improve the work experience when accompanied by adequate preparation and support. Technology adoption scores increased by 28.5% from 68.4 ± 12.3 to 87.9 ± 8.7 (mean increase 19.5 points, 95% CI: 15.2–23.8, Cohen’s d = 1.84 with 95% CI: 1.51–2.17, *p* < 0.001), indicating strong user engagement with the system.

Quality and safety outcomes demonstrated consistent improvement patterns that directly impacted patient care and organizational performance. Safety incident rates decreased by 43.8% from 3.2 ± 1.1 to 1.8 ± 0.7 per 1,000 procedures (mean decrease 1.4 events per 1,000 procedures, 95% CI: 0.9–1.9, Cohen’s d = 1.67 with 95% CI: 1.34–2.00, *p* < 0.001), representing significant enhancement in patient safety through proactive risk identification and mitigation. The quality of care index increased by 16.9% from 79.1 ± 6.8 to 92.5 ± 4.1 (with a mean increase of 13.4, 95% CI: 11.2–15.6, Cohen’s d = 2.18, 95% CI: 1.82–2.54, *p* < 0.001), indicating substantial improvements in overall care delivery quality as measured through validated quality assessment tools.

### 3.4 Predictive modeling and multivariate analysis

Multivariate regression analysis identified predictors of overall system performance and explained 84.7% of the variance in system performance outcomes [*R*^2^ = 0.847, adjusted *R*^2^ = 0.842, *F* (10,336) = 186.0, *p* < 0.001]. Table 4 presents the regression results, including raw and FDR-adjusted *p* values. These findings should be interpreted as associations within the integrated implementation context rather than as evidence of isolated causal effects.

**Table 4:** Multivariate regression analysis: predictors of system performance (*R*^2^ = 0.847, adjusted *R*^2^ = 0.842).

Predictor variable	Beta coefficient	Standard error	*t*-statistic	*p*-value (raw/FDR)	VIF	Partial *R*^2^
Equipment age (years)	−0.34	0.08	−4.25	<0.001/<0.001	1.23	0.154
Staff training hours (cumulative)	0.67	0.12	5.58	<0.001/<0.001	1.45	0.267
Technology integration score	0.58	0.11	5.27	<0.001/<0.001	1.67	0.238
Maintenance protocol adherence (%)	0.73	0.09	8.11	<0.001/<0.001	1.34	0.412
Interdisciplinary communication index	0.45	0.10	4.50	<0.001/<0.001	1.56	0.176
Workload complexity factor	−0.29	0.07	−4.14	<0.001/<0.001	1.28	0.147
Previous system experience (months)	0.41	0.09	4.56	<0.001/<0.001	1.41	0.182
Organizational readiness score	0.62	0.11	5.64	<0.001/<0.001	1.52	0.273
Leadership support index	0.48	0.10	4.80	<0.001/<0.001	1.39	0.198
Baseline performance level	0.35	0.08	4.38	<0.001/<0.001	1.29	0.165

VIF, Variance Inflation Factor; All VIF values <2.0 indicate absence of multicollinearity concerns; Model F(10,336) = 186.0, *p* < 0.001; FDR-adjusted p values were calculated using the Benjamini-Hochberg procedure.

Maintenance Protocol Adherence emerged as the dominant predictor of system performance (beta = 0.73, SE = 0.09, *t* = 8.11, raw and FDR-adjusted *p* < 0.001, Partial *R*^2^ = 0.412), accounting for 41.2% of the explained variance. This finding indicates that consistent application of evidence-based maintenance procedures formed the operational foundation for improved system performance and reinforces that the observed improvements cannot be attributed to AI alone.

Staff Training Hours demonstrated substantial predictive power (*β* = 0.67, SE = 0.12, *t* = 5.58, *p* < 0.001, Partial *R*^2^ = 0.267), explaining 26.7% of the unique variance. This relationship indicates that comprehensive education and skill development were central to implementation success, with greater staff preparation associated with stronger performance gains (16).

Technology Integration Score showed strong positive relationships with performance (*β* = 0.58, SE = 0.11, *t* = 5.27, *p* < 0.001, Partial *R*^2^ = 0.238), highlighting the critical importance of seamless integration between AI systems and existing technological infrastructure. This finding suggests that successful AI implementation requires substantial attention to technical compatibility, interface design, and system interoperability rather than simply adding new technologies to existing environments.

Organizational factors demonstrated substantial influence on implementation success, with the Organizational Readiness Score (*β* = 0.62, SE = 0.11, *t* = 5.64, *p* < 0.001, Partial *R*^2^ = 0.273) and Leadership Support Index (*β* = 0.48, SE = 0.10, *t* = 4.8, *p* < 0.001, Partial *R*^2^ = 0.198) showing significant predictive power. These findings emphasize the fundamental importance of institutional commitment, change management capabilities, and executive support in achieving successful AI-enhanced quality improvement initiatives.

**Figure 4:**
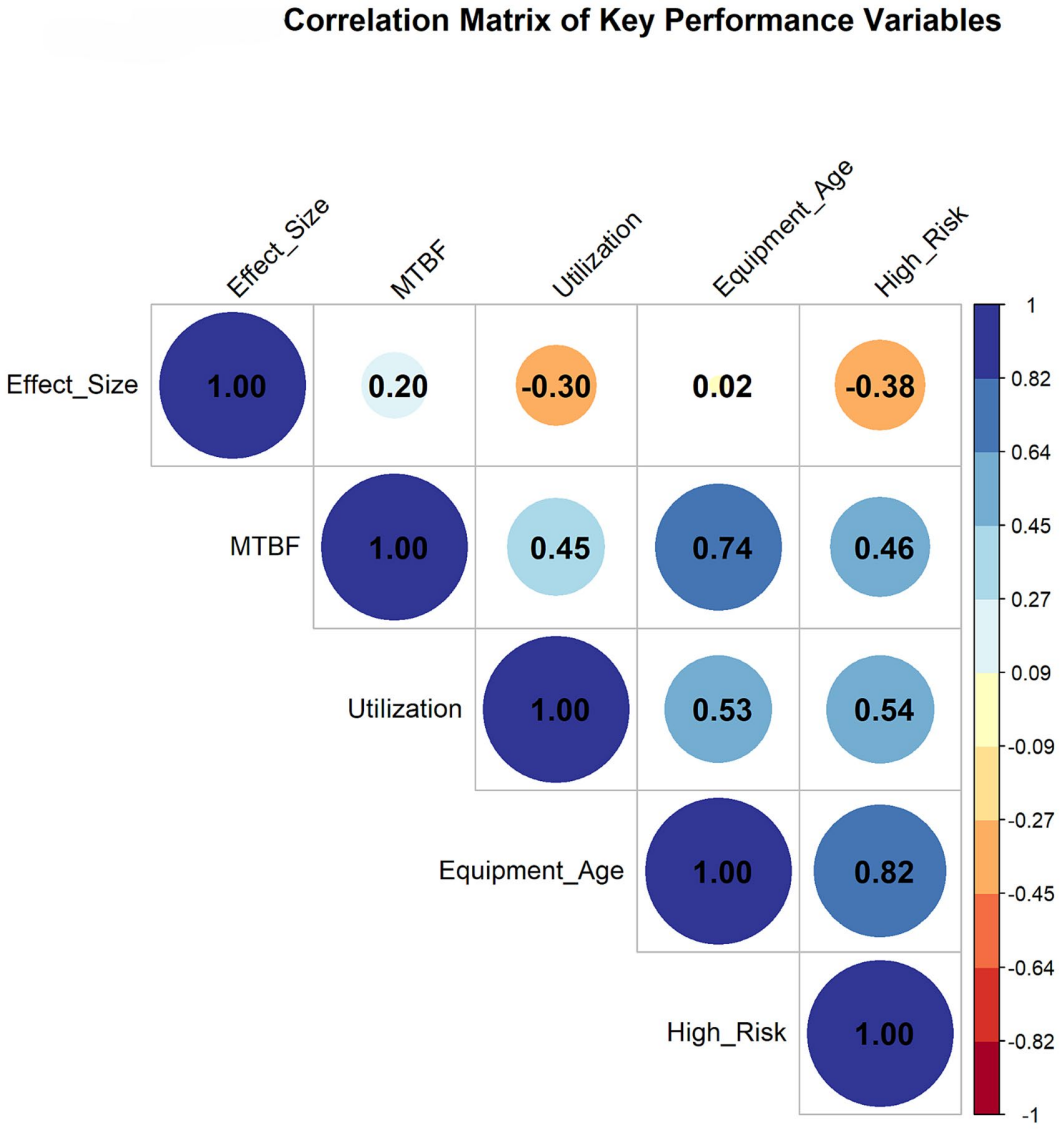
Correlation matrix of key performance variables. Correlation analysis showing relationships between key performance variables, including equipment age, high-risk classification, utilization rates, MTBF, and effect sizes. The matrix uses color intensity and circle sizes to represent correlation strength and direction, with hierarchical clustering applied to group related variables. Only correlations with absolute values greater than 0.3 are displayed to highlight meaningful relationships.

Negative predictors provided important insights into implementation challenges, with Equipment Age showing significant negative relationships (*β* = −0.34, SE = 0.08, *t* = −4.25, *p* < 0.001, Partial *R*^2^ = 0.154), indicating that older equipment posed greater challenges for AI-enhanced optimization, likely due to limited sensor capabilities and integration constraints. Workload Complexity Factor also demonstrated a negative relationship (*β* = −0.29, SE = 0.07, *t* = −4.14, *p* < 0.001, Partial *R*^2^ = 0.147), suggesting that highly complex operational environments require additional adaptation and customization for optimal AI performance. Figure 4 presents the correlation matrix revealing the relationships between the key performance variables, including the equipment characteristics and effect sizes.

### 3.5 Economic analysis and return on investment

A comprehensive cost-effectiveness analysis was conducted from the hospital operational perspective using 2024 Chinese Yuan (CNY) as the currency, with no discounting applied for the first-year analysis. The analysis estimated an initial implementation investment of ¥3,152,000 and annual recurrent operating costs of ¥703,000, resulting in total first-year non-throughput costs of ¥3,855,000. The primary economic calculation examined direct cost savings and indirect benefits separately from potential revenue enhancement attributable to increased surgical throughput. Table 5 presents the revised economic analysis across investment categories.

**Table 5:** Cost-effectiveness analysis, primary non-throughput return on investment, and revenue sensitivity scenarios (Chinese Yuan, ¥).

Cost category	Initial cost (¥)	Annual cost (¥)	Direct savings (¥)	Indirect benefits^a^ (¥)	Net benefit year 1 (¥)	ROI (%)	Payback period (months)
Implementation costs	912,000	0	0	0	−912,000	N/A	N/A
Staff training and development	639,000	165,000	481,000	323,000	0	0.0	12.0
Technology integration	1,120,000	86,000	962,000	560,000	316,000	26.2	9.5
System maintenance (Annual)	0	323,000	639,000	481,000	797,000	246.7	3.5
Quality monitoring tools	481,000	129,000	323,000	244,000	−43,000	−7.0	12.9
Total investment	3,152,000	703,000	2,405,000	1,608,000	158,000	4.1	11.5

Additional revenue generation		
Revenue category	Annual amount (¥)	Calculation basis
Throughput revenue, 100% realization	16,100,000	1,914 additional cases × ¥8,420 net contribution margin per case
Throughput revenue, 75% realization	12,075,000	1,436 additional cases × ¥8,420 net contribution margin per case
Throughput revenue, 50% realization	8,050,000	957 additional cases × ¥8,420 net contribution margin per case
Contribution margin, 20% lower	12,880,000	1,914 additional cases × ¥6,736 net contribution margin per case
Contribution margin, 20% higher	19,320,000	1,914 additional cases × ¥10,104 net contribution margin per case

^a^Indirect benefits are estimated values based on industry benchmarks for staff turnover costs, liability insurance premium reductions, and reputation enhancement. Primary non-throughput ROI was calculated as (Direct Savings + Indirect Benefits–Initial Cost–Annual Cost)/(Initial Cost + Annual Cost) × 100, excluding separate surgical throughput revenue. Payback period was calculated as (Initial Cost + Annual Cost)/(Direct Savings + Indirect Benefits) × 12 months. Surgical throughput revenue is presented as a separate sensitivity scenario because it depends on institutional capacity to convert recovered OR time into additional completed cases.

**Figure 5:**
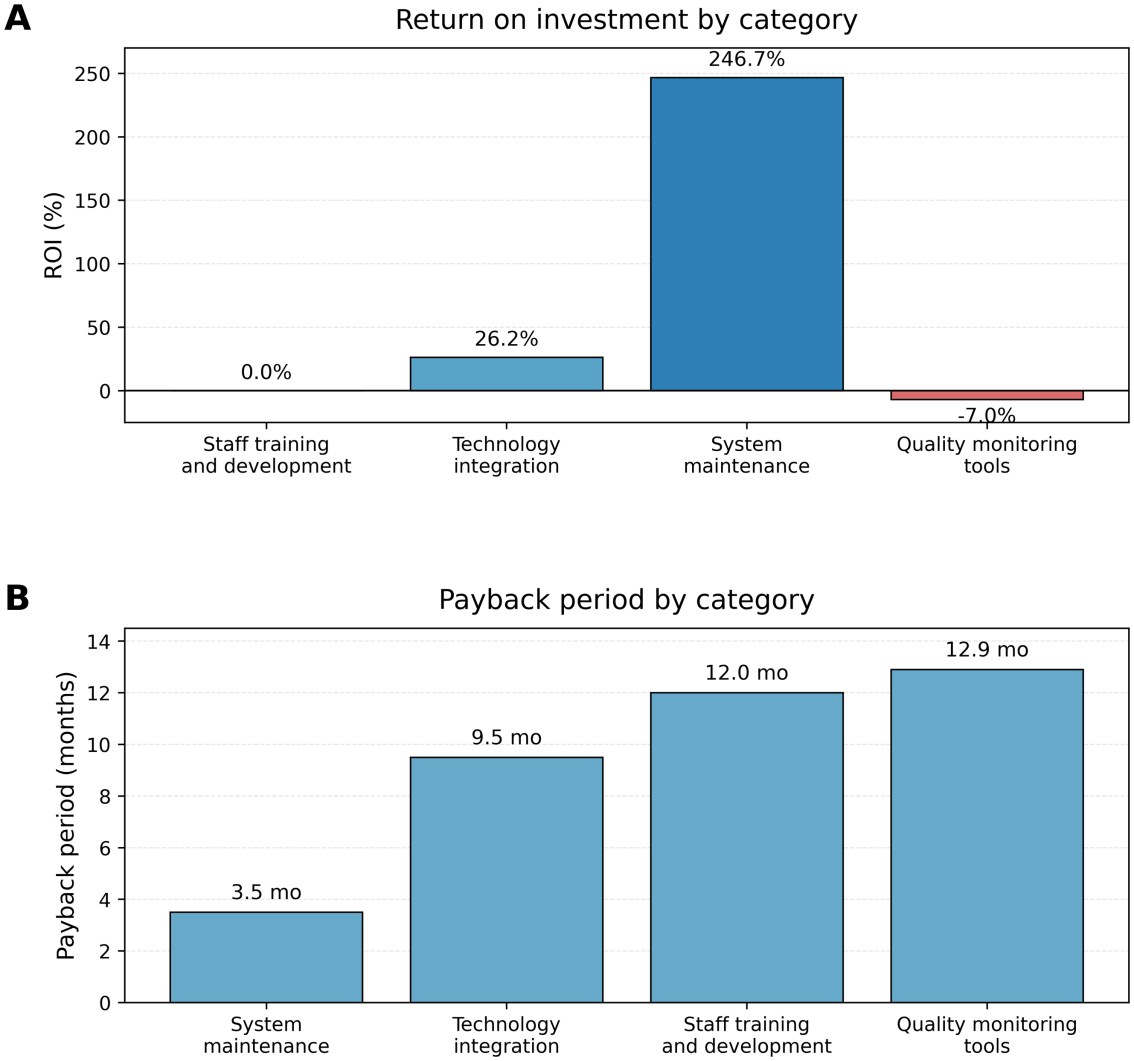
Economic analysis and return on investment. Financial performance visualization across investment categories. Panel **(A)** shows primary non-throughput return on investment percentages by category, with color coding based on ROI magnitude. Panel **(B)** displays payback periods in months, sorted from shortest to longest. Category panels exclude the implementation-cost row because it represents initial infrastructure investment without direct category-level returns, whereas the total calculation includes implementation costs and annual recurrent costs. Values represent first-year projections based on observed improvements and the stated assumptions of the hospital operational economic model.

Direct cost savings totaling ¥2,405,000 annually were estimated during the first operational year, with Technology Integration generating the largest savings category at ¥962,000 annually through reduced equipment downtime, improved utilization rates, and decreased emergency repair costs. These savings reflected measurable reductions in invoiced emergency maintenance costs, overtime wage payments, and equipment rental expenses associated with improved operational performance. Figure 5 visualizes financial performance across investment categories, including primary non-throughput return on investment percentages and payback periods.

System Maintenance achieved the highest category-level estimated primary return on investment at 246.7%, with a payback period of 3.5 months, generating ¥639,000 in direct annual savings and ¥481,000 in indirect benefits through predictive maintenance capabilities that reduced emergency repairs and supported equipment lifecycle management. This estimate is closely linked to the predictive failure algorithm’s 92.8% accuracy and should be interpreted in relation to the hospital’s baseline maintenance burden and equipment inventory.

Staff Training and Development required substantial upfront investment (¥639,000) and ongoing annual costs (¥165,000), with estimated direct savings and indirect benefits totaling ¥804,000. Under the revised first-year formula, this category reached cost neutrality in the first year, corresponding to 0.0% primary ROI and a 12.0-month payback period. These estimates reflected reduced recruitment and training costs for replacement staff, decreased overtime expenses through improved efficiency, and enhanced job satisfaction associated with improved retention.

Quality Monitoring Tools generated ¥323,000 in direct savings and ¥244,000 in indirect benefits through improved quality outcomes, reduced safety incidents, and enhanced regulatory compliance. Because the combined first-year cost for this category was ¥610,000, the revised category-level net benefit was negative in the first year (¥-43,000), corresponding to a primary ROI of −7.0% and a payback period of 12.9 months. This category may therefore require a longer time horizon before producing a positive standalone financial return.

Operational improvements were monetized separately from direct cost savings through the calculation of incremental surgical cases potentially enabled by reduced room turnover time and improved equipment availability. The 9.5-min reduction in average turnover time yielded an estimated 1,914 additional surgical cases annually across the 28 SRRSH operating rooms if recovered time were fully converted into additional completed cases. These additional cases were valued using a net contribution margin per case of ¥8,420 obtained from hospital finance department records, representing average incremental revenue minus variable costs per surgical procedure. This produced an estimated annual revenue enhancement of ¥16.1 million under the full-capacity conversion scenario. This projected revenue is reported separately and is not included in the primary non-throughput ROI calculation.

Indirect benefits totaling ¥1,608,000 annually represent estimated values based on industry benchmarks for staff turnover costs, professional liability insurance premium reductions, and organizational reputation enhancement. These estimates include improvements in staff productivity, patient satisfaction, reduced liability exposure, and enhanced organizational reputation. Because these components are more difficult to quantify precisely than direct maintenance and overtime costs, they should be interpreted as scenario-based estimates rather than guaranteed financial returns.

Revenue effects from increased surgical throughput (¥16.1 million annually under full-capacity conversion) are presented separately from direct cost savings (maintenance, overtime, and quality improvement totaling ¥2.4 million annually) to distinguish expense reductions from potential revenue enhancement. The primary non-throughput ROI reported in Table 5 is 4.1%, based on direct savings and indirect benefits after subtracting both initial implementation costs and annual recurrent operating costs, while excluding the separate surgical throughput revenue scenario. Direct cost savings alone did not fully offset total first-year cost; the positive first-year ROI was achieved only when indirect benefits were included.

Key assumptions underlying the economic model included stable surgical demand, the ability to staff and schedule additional surgical capacity, no major change in case mix or reimbursement during the study year, constant contribution margins, and no major fixed-cost expansion required to accommodate increased throughput. One-way sensitivity scenarios were added for 50, 75, and 100% realization of additional surgical capacity and for a 20% decrease or increase in net contribution margin. These assumptions may not hold in smaller hospitals, institutions with lower baseline utilization, constrained staffing, different payer structures, or limited capacity to convert time savings into additional cases. Accordingly, the ROI estimates should be viewed as institution-specific projections rather than universally transferable economic effects.

### 3.6 Artificial intelligence performance assessment

The AI components of the three-dimensional integrated quality model demonstrated strong performance across internal assessment metrics, supporting the technical feasibility of AI-enhanced quality management in this operating room environment. Predictive maintenance algorithms achieved 92.8% accuracy (95% CI: 89.4–95.3%) in predicting equipment failures 48–72 h in advance, with a precision of 89.3%, recall of 91.7%, F1-score of 90.5%, and area under the receiver operating characteristic curve (AUC-ROC) of 0.94 (95% CI: 0.91–0.97).

The predictive failure target was defined as equipment failure within 48–72 h, where failures comprised unplanned service interruptions lasting more than 15 min that required biomedical technician intervention. The temporal assessment strategy employed training on data from months 1 through 8 and validation on months 9 through 12, with equipment IDs held across splits to reduce information leakage within the institutional dataset. These analyses assessed internal predictive performance and do not constitute external validation.

The false-positive rate was maintained at 6.2%, indicating an effective balance between sensitivity and specificity that minimized unnecessary maintenance interventions while capturing true failure events. The positive predictive value reached 89.3%, indicating that approximately 9 out of 10 predictive alerts corresponded to actual impending failures, enabling confident resource allocation for proactive maintenance activities. Calibration analysis demonstrated excellent agreement between predicted and observed failure probabilities, with a Brier score of 0.082 and Hosmer-Lemeshow chi-square of 8.34 (*p* = 0.40), indicating good calibration across the full range of predicted probabilities. The calibration plots presented in Supplementary Figure S1 show a strong concordance between the predicted and observed failure rates across the probability deciles.

For the operational efficiency dimension, real-time quality assessment algorithms achieved 87.3% accuracy (95% CI: 84.1–90.2%) in predicting room turnover delays exceeding 5 min beyond the scheduled time, with a precision of 84.1% and recall of 89.6%. Dynamic resource allocation optimization demonstrated a 23.7% improvement in equipment utilization prediction accuracy compared to baseline rule-based scheduling algorithms (baseline accuracy 68.4%, AI-enhanced accuracy 84.6%, *p* < 0.001).

For the staff performance dimension, internal assessment analyses demonstrated that the workflow integration scoring system achieved strong inter-rater reliability (intraclass correlation coefficient = 0.89, 95% CI: 0.85–0.92) between independent observers and demonstrated criterion evidence through correlation with independent expert performance assessments (*r* = 0.76, *p* < 0.001). The technology adoption prediction model achieved 83.4% accuracy (95% CI: 79.8–86.7%) in identifying staff members who would require additional training support, with sensitivity of 81.2% and specificity of 85.6%.

Anomaly detection algorithms using Isolation Forest and Local Outlier Factor techniques identified 94.3% of actual equipment anomalies while maintaining false positive rates of 8.1%, demonstrating the ability to distinguish meaningful performance deviations from normal operational variation within the SRRSH data environment. Cross-validation analyses showed consistent performance across equipment types, operational contexts, and temporal periods within the study site.

Real-time optimization algorithms processed an average of 2.3 million data points daily from the SRRSH operating room complex, with average response times of 127 milliseconds for predictive alerts and 89 milliseconds for operational optimization recommendations. The system maintained 99.7% uptime throughout the study period, with two brief service interruptions totaling 4.2 h over 12 months.

**Figure 6:**
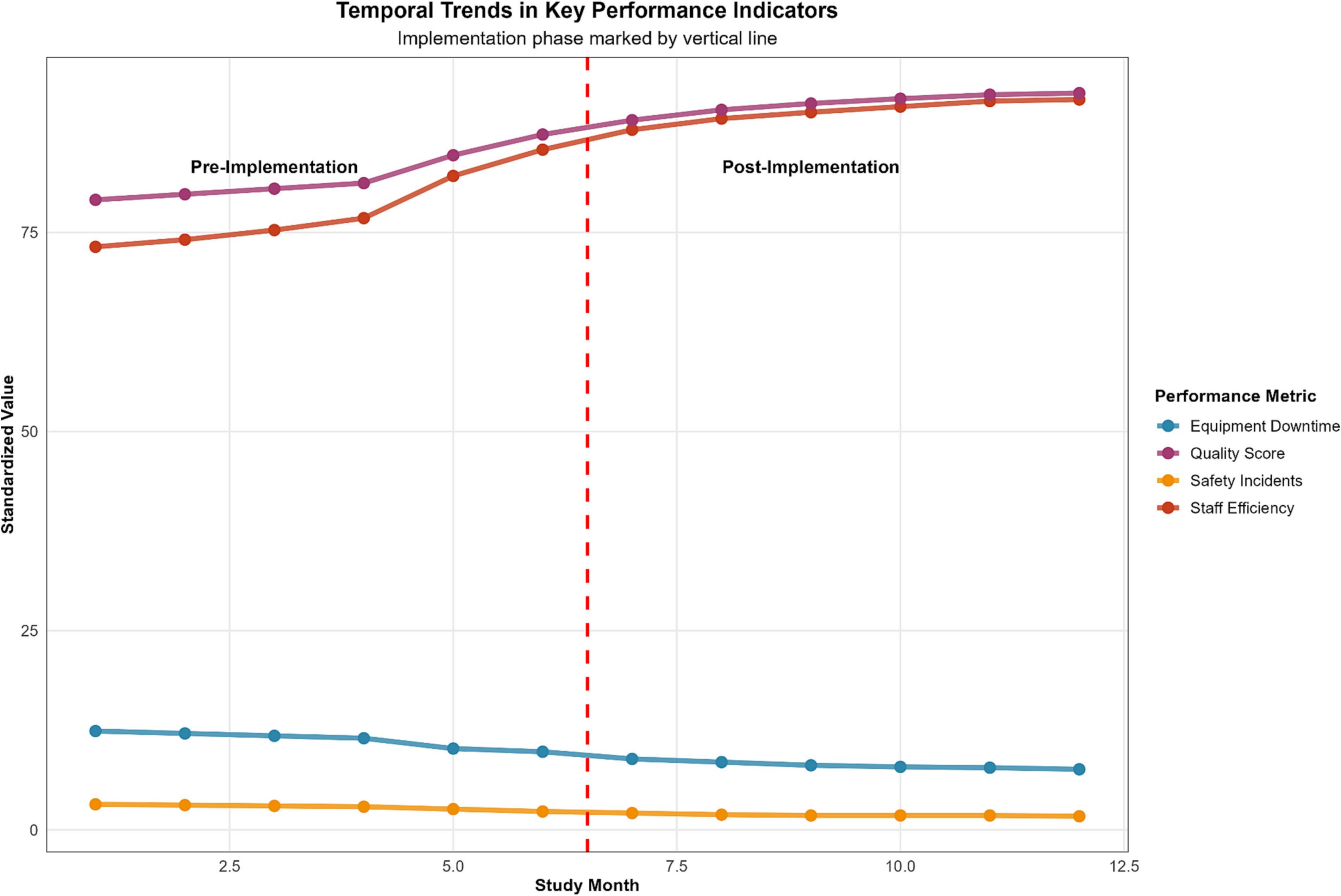
Temporal trends in key performance indicators. Time-series analysis showing monthly performance trajectories for four key indicators throughout the study period. The vertical dashed line at month 6.5 marks the implementation phase transition. Data points represent observed values with smoothed trend lines using LOESS regression and 95% confidence intervals. The visualization demonstrates post-implementation performance trajectories across measured domains.

Model performance improved over time, with accuracy gains of 3.2% for predictive maintenance and 4.7% for operational optimization observed between months 6 and 12 of implementation. These improvements were associated with continuous model retraining using expanding datasets and refined algorithmic parameters. Beyond AI-specific accuracy metrics, broader operational performance also evolved across the study period; Figure 6 shows the temporal trajectories of equipment downtime, quality of care, safety incidents, and staff efficiency, with the implementation phase transition marked at month 6.5.

## 4 Discussion

This prospective single-center study evaluated an integrated AI-enhanced three-dimensional quality model for quality-sensitive indicators in OR management within a major Chinese academic medical center. The principal finding is that implementation of a combined intervention, including AI-enabled prediction, IoT-supported monitoring, staff training, maintenance protocol reinforcement, and workflow optimization, was associated with clinically and operationally meaningful improvements across equipment, room-level, staff-level, and system-level outcomes. The results should therefore be interpreted as evidence supporting the feasibility and potential value of an integrated quality-management strategy, rather than as proof that AI alone caused the observed improvements.

The clinical relevance of the findings lies in the alignment between operational performance and quality-sensitive outcomes. Reductions in equipment downtime and room turnover time may improve schedule reliability, reduce avoidable delays, and increase the availability of surgical resources. The observed decrease in safety incident rates and increase in quality-of-care indices are consistent with the premise that better equipment readiness, clearer workflow coordination, and more timely operational feedback can support safer perioperative care. These findings align with the broader literature describing the potential of AI-supported systems to improve nursing practice and patient care quality when embedded within appropriate clinical governance structures (8).

Staff-related findings are particularly important because technology implementation in healthcare can either support or burden frontline personnel depending on design and organizational context (17). In this study, staff efficiency, satisfaction, and technology adoption scores improved after implementation, suggesting that AI-supported tools may enhance work experience when they reduce equipment disruptions, make resource availability more predictable, and are accompanied by structured training and managerial support. This interpretation is consistent with recent work emphasizing that AI should augment human expertise and maintain human oversight rather than displace clinical judgment (1, 3, 9, 10).

The central methodological contribution of this study is the integration of three elements that are often examined separately: an internally assessed three-dimensional indicator structure, machine learning models assigned to defined operational tasks, and workflow mechanisms that translated model outputs into maintenance, scheduling, and staff development actions. Predictive maintenance extended earlier IoT-based and data-driven equipment management approaches (11, 12) by incorporating multimodal sensor data into a broader quality framework. The model’s value therefore resides not only in algorithmic prediction, but in its capacity to connect predictions to interpretable quality indicators and operational decisions.

The three-dimensional modeling approach helped clarify interdependencies between equipment performance, operational processes, and human factors. Moderate correlations among the three dimensions supported the theoretical premise that these domains are related yet distinct. This structure may be useful for OR leaders because it prevents improvements in one area from being interpreted in isolation. For example, maintenance protocol adherence and technology integration were both strongly associated with system performance, indicating that technical prediction and disciplined operational execution were mutually reinforcing rather than interchangeable.

The economic findings provide a hospital-level estimate of potential financial value, but they require cautious interpretation. Direct cost savings were grounded in maintenance, overtime, and quality-related cost data, whereas revenue enhancement depended on the assumption that improved turnover time and equipment availability could be converted into additional completed surgical cases. We therefore report the primary non-throughput ROI separately from revenue-enhancement sensitivity scenarios. The revised non-throughput calculation produced a modest first-year ROI, indicating that the implementation may be financially sustainable under the observed assumptions but should not be interpreted as a universally high-return intervention. The estimated ROI is most applicable to institutions with stable surgical demand, sufficient staffing, and the operational capacity to use recovered time. Institutions with different case mixes, reimbursement systems, staffing constraints, or baseline utilization may experience smaller or different financial effects.

Implementation predictors provide practical guidance for institutions considering similar programs. Maintenance protocol adherence, staff training hours, technology integration, organizational readiness, and leadership support were all associated with stronger system performance. These findings underscore that AI implementation is not solely a technical project. It requires reliable data infrastructure, clear governance, staff preparation, and leadership alignment. In less resourced settings, these prerequisites may require staged implementation before the full benefits of AI-enabled quality management can be realized.

This study has several limitations. The single-center design at a well-resourced tertiary academic hospital limits generalizability to smaller hospitals, rural settings, or institutions with different technological infrastructure and staffing models. The 12-month duration provides useful evidence regarding implementation and early sustainability but does not establish long-term performance stability or delayed unintended consequences. The study was powered for moderate effects but observed very large effect sizes; therefore, statistical significance should be interpreted alongside absolute effect magnitude, baseline trend assessment, and clinical plausibility. Most importantly, the pragmatic pre-post design cannot isolate the AI system’s independent contribution from concurrent improvements in training, protocol reinforcement, leadership attention, and workflow redesign. The most accurate interpretation is that the AI-enhanced system, when integrated with evidence-based protocols and comprehensive training, supported and accelerated improvements while enabling capabilities, such as predictive maintenance alerts and real-time optimization recommendations, that would be difficult to reproduce through conventional methods alone (7, 13).

Future research should use controlled or stepped-wedge designs to compare AI-enhanced quality-management programs with conventional improvement programs and thereby quantify AI-specific contributions. Multicenter studies across diverse Chinese and international healthcare settings are needed to examine transferability, infrastructure requirements, and context-dependent implementation barriers. Longer follow-up is also required to assess durability, model drift, cost stability, and ethical considerations related to transparency, data governance, staff autonomy, and equity (14, 15).

In conclusion, implementation of an AI-enhanced three-dimensional integrated quality model, when combined with evidence-based protocols, staff training, and workflow redesign, was associated with substantial improvements in OR performance at a single tertiary academic medical center. The observed reductions in equipment downtime, room turnover time, and safety incidents, together with improvements in staff efficiency and satisfaction, support the practical value of an integrated quality-management framework. Because the intervention was multifactorial and evaluated without a concurrent control group, these findings should be regarded as promising and context-dependent. Further controlled multicenter studies are needed before the model can be recommended as a broadly generalizable approach for healthcare organizations with different resources, workflows, and implementation capacities.

## Data Availability

The raw data supporting the conclusions of this article will be made available by the authors, without undue reservation.
