# Supplementary material for: Development and preliminary evaluation of an AI-enhanced three-dimensional integrated quality model for quality-sensitive indicators in operating room management: a prospective single-center study
Source: Front Med (Lausanne). 2026 May 26;13:1830302. doi: 10.3389/fmed.2026.1830302 (PMC13247684; doi:10.3389/fmed.2026.1830302)
Supplement: Supplementary file 1 [file Supplementary_file_1.docx]

**Supplementary Table S1: Complete Quality Indicator Specifications**

**Equipment Management Dimension (X-Axis)**

| **Indicator Name** | **Operational Definition** | **Measurement Unit/Range** | **Directionality** | **Data Source** | **Delphi Weight** | **Validation Evidence** |
| --- | --- | --- | --- | --- | --- | --- |
| **Equipment Utilization Efficiency Index** | Ratio of actual operational time to scheduled available time. Formula: (Actual operational hours / Scheduled available hours) × 100 | Percentage; 0-100% | Higher = Better | IoT sensor networks with 1-minute logging intervals | 0.45 | Criterion validity: r=0.82 (p<0.001) with manually recorded utilization logs; Test-retest ICC=0.91 (95% CI: 0.87-0.94) |
| **Maintenance Response Time** | Mean interval from equipment failure alert to completion of repair, measured in hours | Hours; Range 0-24 hours | Lower = Better | Automated failure detection timestamps integrated with computerized maintenance management system | 0.62 | Inter-rater reliability: ICC=0.94 (95% CI: 0.91-0.96) between automated and manual timing; Concurrent validity: r=0.88 with supervisor ratings |
| **Predictive Failure Algorithm Accuracy** | Proportion of correctly predicted equipment failures within 48-72 hour prediction window. Failures defined as unplanned service interruptions >15 minutes requiring technician intervention | Percentage; 0-100% | Higher = Better | Machine learning prediction logs validated against actual failure events from maintenance database | 0.71 | Cross-validation accuracy: 92.8% (95% CI: 89.4-95.3%); AUC-ROC=0.94; Temporal validation with holdout sets |
| **Multi-Modal Performance Index** | Composite measure integrating: (1) Temperature stability (coefficient of variation), (2) Vibration patterns (amplitude deviation from baseline), (3) Energy consumption efficiency (actual vs. expected ratio). Formula: Weighted average of normalized sub-metrics with equal weighting | Score; 0-100 | Higher = Better | IoT sensors capturing thermal (±0.1°C accuracy), mechanical (±0.01 mm/s vibration), and electrical (±1W power) parameters | 0.56 | Internal consistency: Cronbach's α=0.91; Convergent validity: r=0.84 with expert equipment condition ratings; Test-retest ICC=0.87 |

**Operational Efficiency Dimension (Y-Axis)**

| **Indicator Name** | **Operational Definition** | **Measurement Unit/Range** | **Directionality** | **Data Source** | **Delphi Weight** | **Validation Evidence** |
| --- | --- | --- | --- | --- | --- | --- |
| **Dynamic Resource Allocation Score** | Composite measure of: (1) Scheduling optimization (variance between actual and optimal schedules), (2) Equipment assignment efficiency (equipment-procedure matching appropriateness), (3) Staff allocation effectiveness (skill-task alignment). Calculated by comparing algorithm-generated recommendations to actual utilization patterns | Score; 0-100 | Higher = Better | Operating room management system with real-time case tracking and staff scheduling database | 0.42 | Convergent validity: r=0.74 (p<0.001) with expert efficiency ratings; Predictive validity: β=0.68 for predicting throughput; Internal consistency: α=0.86 |
| **Real-time Quality Assessment** | Continuous monitoring score aggregating: (1) Process compliance (adherence to surgical safety checklist), (2) Timing adherence (variance from scheduled milestones), (3) Safety protocol completion (percentage of required safety steps completed). Measured through EHR documentation completeness | Score; 0-100 | Higher = Better | Integrated electronic health record system with automated process tracking and timestamp validation | 0.35 | Criterion validity: r=0.79 (p<0.001) with manual quality audits; Inter-rater reliability: ICC=0.83; Content validity index=0.92 from expert panel |
| **Room Turnover Optimization Index** | Efficiency of room preparation processes measured through actual completion time relative to case complexity-adjusted benchmarks. Accounts for: case type (minor/major surgery), cleaning requirements (level 1-4), equipment setup needs (standard/complex) | Normalized efficiency score; 0-100 | Higher = Better | Automated room status tracking system with RFID sensors and case scheduling integration | 0.38 | Test-retest reliability: ICC=0.88 across consecutive cases; Concurrent validity: r=0.82 with time-motion study measurements; Sensitivity to change: d=1.2 |
| **Patient Flow Efficiency Metric** | Smoothness of patient movement through perioperative phases, quantified by coefficient of variation in timing for: (1) Pre-operative preparation, (2) Intra-operative duration relative to scheduled time, (3) Post-operative transfer to recovery. Lower variance indicates better flow | Coefficient of variation; 0-50% (inverted for scoring) | Lower variance = Better | EHR timestamps: admission, room entry, incision, closure, room exit, recovery admission; Patient tracking system | 0.53 | Concurrent validity: r=0.81 (p<0.001) with patient satisfaction scores; Predictive validity: r=-0.76 with delay-related complaints; Test-retest ICC=0.85 |

**Staff Performance Dimension (Z-Axis)**

| **Indicator Name** | **Operational Definition** | **Measurement Unit/Range** | **Directionality** | **Data Source** | **Delphi Weight** | **Validation Evidence** |
| --- | --- | --- | --- | --- | --- | --- |
| **Staff Workflow Integration Score** | Composite measure combining: (1) Task completion time efficiency (standardized task duration vs. benchmark), (2) Workflow interruption frequency (interruptions per hour), (3) Technology interaction smoothness (system navigation clicks per task). Assessed through structured observation using standardized time-motion protocols | Score; 0-100 | Higher = Better | Direct observation by trained observers using validated time-motion study instruments; Electronic system usage analytics logs | 0.36 | Inter-rater reliability: ICC=0.89 (95% CI: 0.85-0.92); Internal consistency: Cronbach's α=0.92; Criterion validity: r=0.81 with supervisor performance ratings |
| **Technology Adoption Score** | Proficiency and engagement with AI-enhanced systems measured through: (1) Technology Acceptance Model (TAM) questionnaire (perceived usefulness, ease of use, behavioral intention), (2) Objective usage metrics (system login frequency, feature utilization breadth, recommendation adherence rates) | Composite score; 0-100 | Higher = Better | Validated TAM instrument (Chinese version, 18 items, 5-point Likert) combined with electronic system analytics tracking user interactions | 0.48 | Internal consistency: Cronbach's α=0.90 for TAM subscales; Convergent validity: r=0.78 with actual system usage frequency; Test-retest reliability: ICC=0.86 over 2-week interval |
| **Communication Effectiveness Index** | Quality of interdisciplinary communication assessed through structured observation of: (1) Pre-operative briefings, (2) Intra-operative handoffs, (3) Post-operative debriefings. Scored using WHO Surgical Safety Checklist Communication Assessment Tool on standardized rubrics evaluating information completeness (0-4), clarity (0-4), and team engagement (0-4) | Score; 0-100 (normalized from 0-12 raw scale) | Higher = Better | Direct observation with validated WHO communication assessment instruments; Communication quality logs maintained by charge nurses | 0.37 | Inter-rater reliability: ICC=0.87 (95% CI: 0.83-0.91); Content validity: validated by WHO guidelines; Criterion validity: r=0.73 with safety culture survey scores |
| **Professional Development Progress** | Quarterly competency assessment using hospital-validated evaluation tools measuring: (1) Technical skills (equipment operation, emergency response), (2) Critical thinking (clinical decision-making, problem-solving), (3) Professional behaviors (teamwork, communication, leadership). Progression tracked relative to individualized learning plans with standardized competency rubrics | Competency achievement percentage; 0-100% | Higher = Better | Structured competency evaluation records using hospital-validated assessment forms; Training completion database; Annual performance review documentation | 0.27 | Content validity index: 0.94 from 12-member expert panel review; Inter-rater reliability: ICC=0.82 between evaluators; Criterion validity: r=0.79 with annual performance ratings; Internal consistency: α=0.88 |

**Excluded Indicators and Rationale**

| **Excluded Indicator** | **Dimension** | **Reason for Exclusion** | **Statistical Evidence** |
| --- | --- | --- | --- |
| Equipment Lifecycle Stage Classification | Equipment | Excessive multicollinearity with Equipment Age | VIF=8.7; r=0.92 with Equipment Age |
| Maintenance Cost per Equipment Hour | Equipment | Excessive multicollinearity with Maintenance Response Time | VIF=9.2; r=0.89 with Maintenance Response Time |
| Supply Chain Integration Efficiency | Operational | Excessive missing data | 34.8% missing values; imputation deemed unreliable |
| Team Cohesion Climate Score | Staff | Poor test-retest reliability | ICC=0.52 (95% CI: 0.38-0.64); α=0.68 |

**Data Collection Schedule and Frequency**

| **Indicator** | **Collection Frequency** | **Assessment Method** | **Responsible Personnel** |
| --- | --- | --- | --- |
| Equipment Management Indicators | Continuous (1-minute intervals) | Automated IoT sensors | Biomedical engineering technicians (calibration and validation) |
| Operational Efficiency Indicators | Real-time to Daily | Automated EHR/system logs | OR coordinators (data verification) |
| Staff Performance Indicators | Weekly to Quarterly | Mixed: Observational assessments (weekly), Surveys (quarterly), Competency evaluations (quarterly) | Nurse managers and trained observers |

**Weighting Methodology**

Delphi weights were derived through a modified three-round Delphi process:

**Round 1:** 28 expert panelists independently rated importance of each indicator on 0-10 scale and provided dimension assignment recommendations

**Round 2:** Panelists received anonymized aggregate results and were asked to re-rate indicators considering group feedback; Indicators achieving ≥80% consensus on importance ratings (±1 point) and dimension assignment advanced

**Round 3:** Final weight calibration through pairwise comparison matrices within each dimension; Analytic Hierarchy Process (AHP) used to derive final normalized weights ensuring sum of weights within each dimension equals optimal representation

**Inter-rater Agreement:** Kendall's W=0.82 (p<0.001) for importance rankings; Fleiss' κ=0.78 for dimension assignments

**Notes**

All indicators were validated during a 3-month pilot phase (October-December 2023) prior to main study implementation. Validation procedures included criterion validity assessment against gold-standard measurements, inter-rater reliability testing with independent observers, test-retest reliability evaluation over appropriate time intervals, and internal consistency analysis for multi-item composite measures.

Missing data rates for all retained indicators were <5% during the pilot phase and <3% during the main study period, with missingness occurring completely at random (Little's MCAR test: χ²=47.3, p=0.52).

Indicator performance was monitored monthly throughout the study with quality control checks for data completeness, range validation, and outlier detection using automated statistical process control methods.

**Supplementary Table S2: Confirmatory Factor Analysis Results**

**Model Fit Indices for Three-Dimensional Structure**

| **Fit Index** | **Value** | **Threshold for Good Fit** | **Interpretation** |
| --- | --- | --- | --- |
| **Chi-square (χ²)** | 110.72 | - | - |
| **Degrees of freedom (df)** | 51 | - | - |
| **χ²/df ratio** | 2.14 | <3.0 (acceptable); <2.0 (excellent) | Acceptable fit |
| **Comparative Fit Index (CFI)** | 0.96 | >0.95 (excellent); >0.90 (acceptable) | Excellent fit |
| **Tucker-Lewis Index (TLI)** | 0.94 | >0.95 (excellent); >0.90 (acceptable) | Acceptable fit |
| **Root Mean Square Error of Approximation (RMSEA)** | 0.048 | <0.06 (good); <0.08 (acceptable) | Good fit |
| **RMSEA 90% Confidence Interval** | 0.041 - 0.055 | Lower bound <0.05; Upper bound <0.08 | Good fit |
| **Standardized Root Mean Square Residual (SRMR)** | 0.039 | <0.08 (good); <0.10 (acceptable) | Good fit |
| **Akaike Information Criterion (AIC)** | 9,847.3 | Lower is better (for model comparison) | - |
| **Bayesian Information Criterion (BIC)** | 10,124.8 | Lower is better (for model comparison) | - |

*Model estimation: Maximum likelihood with robust standard errors (MLR); Sample size: N=4,164 observations (equipment indicators), N=1,872 observations (staff indicators); Missing data: <3%, handled with Full Information Maximum Likelihood (FIML)*

**Factor Loadings (Standardized)**

**Equipment Management Dimension (X-Axis)**

| **Indicator** | **Standardized Loading** | **Standard Error** | **Z-value** | **p-value** | **R²** |
| --- | --- | --- | --- | --- | --- |
| Equipment Utilization Efficiency Index | 0.71 | 0.04 | 17.75 | <0.001 | 0.50 |
| Maintenance Response Time | 0.78 | 0.04 | 19.50 | <0.001 | 0.61 |
| Predictive Failure Algorithm Accuracy | 0.89 | 0.03 | 29.67 | <0.001 | 0.79 |
| Multi-Modal Performance Index | 0.84 | 0.03 | 28.00 | <0.001 | 0.71 |

**Operational Efficiency Dimension (Y-Axis)**

| **Indicator** | **Standardized Loading** | **Standard Error** | **Z-value** | **p-value** | **R²** |
| --- | --- | --- | --- | --- | --- |
| Dynamic Resource Allocation Score | 0.67 | 0.05 | 13.40 | <0.001 | 0.45 |
| Real-time Quality Assessment | 0.84 | 0.03 | 28.00 | <0.001 | 0.71 |
| Room Turnover Optimization Index | 0.76 | 0.04 | 19.00 | <0.001 | 0.58 |
| Patient Flow Efficiency Metric | 0.81 | 0.03 | 27.00 | <0.001 | 0.66 |

**Staff Performance Dimension (Z-Axis)**

| **Indicator** | **Standardized Loading** | **Standard Error** | **Z-value** | **p-value** | **R²** |
| --- | --- | --- | --- | --- | --- |
| Staff Workflow Integration Score | 0.91 | 0.02 | 45.50 | <0.001 | 0.83 |
| Technology Adoption Score | 0.86 | 0.03 | 28.67 | <0.001 | 0.74 |
| Communication Effectiveness Index | 0.79 | 0.03 | 26.33 | <0.001 | 0.62 |
| Professional Development Progress | 0.73 | 0.04 | 18.25 | <0.001 | 0.53 |

*All factor loadings significant at p<0.001; R² represents proportion of indicator variance explained by latent factor*

**Inter-Factor Correlations**

|  | **Equipment Management** | **Operational Efficiency** | **Staff Performance** |
| --- | --- | --- | --- |
| **Equipment Management** | 1.00 | - | - |
| **Operational Efficiency** | 0.58*** | 1.00 | - |
| **Staff Performance** | 0.51*** | 0.64*** | 1.00 |

****p<0.001; Correlations are factor correlations (not indicator correlations)*

**Interpretation:** Moderate positive correlations between dimensions support the theoretical premise that dimensions are related yet distinct. Shared variance ranges from 26% (Equipment-Staff: 0.51²=26%) to 41% (Operational-Staff: 0.64²=41%), indicating each dimension captures substantial unique variance while acknowledging meaningful interdependencies.

**Discriminant Validity Assessment**

| **Dimension Pair** | **Factor Correlation (r)** | **Shared Variance (r²)** | **Average Variance Extracted (AVE) - Dimension 1** | **AVE - Dimension 2** | **Discriminant Validity Test** |
| --- | --- | --- | --- | --- | --- |
| Equipment - Operational | 0.58 | 0.34 | 0.65 | 0.60 | **PASS** (r² < both AVEs) |
| Equipment - Staff | 0.51 | 0.26 | 0.65 | 0.68 | **PASS** (r² < both AVEs) |
| Operational - Staff | 0.64 | 0.41 | 0.60 | 0.68 | **PASS** (r² < both AVEs) |

*Discriminant validity confirmed: shared variance between factors is less than average variance extracted by each factor, demonstrating that dimensions are empirically distinct*

**Alternative Model Comparisons**

| **Model** | **χ²** | **df** | **χ²/df** | **CFI** | **TLI** | **RMSEA** | **SRMR** | **AIC** | **BIC** | **Δχ² (vs. 3-factor)** | **p-value** |
| --- | --- | --- | --- | --- | --- | --- | --- | --- | --- | --- | --- |
| **Three-factor model (hypothesized)** | 110.72 | 51 | 2.14 | 0.96 | 0.94 | 0.048 | 0.039 | 9,847.3 | 10,124.8 | - | - |
| Single-factor model (all indicators) | 487.35 | 54 | 9.02 | 0.78 | 0.73 | 0.110 | 0.094 | 10,418.2 | 10,673.5 | 376.63 | <0.001 |
| Two-factor model (Equipment vs. Process+Staff) | 254.18 | 53 | 4.79 | 0.87 | 0.84 | 0.082 | 0.067 | 10,087.1 | 10,351.2 | 143.46 | <0.001 |
| Two-factor model (Equipment+Operational vs. Staff) | 298.47 | 53 | 5.63 | 0.84 | 0.80 | 0.089 | 0.073 | 10,131.4 | 10,395.5 | 187.75 | <0.001 |

*Δχ² tests show three-factor model fits significantly better than all alternative structures (all p<0.001)*

**Modification Indices**

**Top 5 Modification Indices (not implemented to preserve theoretical model):**

| **Suggested Modification** | **MI Value** | **Expected Parameter Change** | **Decision** |
| --- | --- | --- | --- |
| Residual correlation: Equipment Utilization ↔ Room Turnover | 14.3 | +0.18 | Not added (cross-dimensional correlation would obscure factor structure) |
| Residual correlation: Maintenance Response ↔ Staff Workflow | 11.7 | -0.14 | Not added (theoretically unjustified) |
| Cross-loading: Technology Adoption → Operational | 9.8 | +0.21 | Not added (violates simple structure) |
| Residual correlation: Predictive Accuracy ↔ Real-time Assessment | 8.4 | +0.12 | Not added (adequate fit without modification) |
| Residual correlation: Communication ↔ Patient Flow | 7.9 | +0.11 | Not added (theoretically unjustified) |

*Model modifications were not implemented as the hypothesized model demonstrated excellent fit (CFI=0.96, RMSEA=0.048) and modifications would compromise theoretical interpretability*

**Reliability and Validity Summary**

| **Dimension** | **Number of Indicators** | **Cronbach's Alpha** | **Composite Reliability (CR)** | **Average Variance Extracted (AVE)** | **Validity Assessment** |
| --- | --- | --- | --- | --- | --- |
| Equipment Management | 4 | 0.88 | 0.89 | 0.65 | Good (AVE>0.50, CR>0.70) |
| Operational Efficiency | 4 | 0.84 | 0.86 | 0.60 | Good (AVE>0.50, CR>0.70) |
| Staff Performance | 4 | 0.90 | 0.91 | 0.68 | Excellent (AVE>0.60, CR>0.90) |
| **Overall Model** | **12** | **0.92** | **0.93** | **0.64** | **Excellent** |

*Composite Reliability (CR) = (Σλ)² / [(Σλ)² + Σ(1-λ²)], where λ = standardized factor loadings*

*Average Variance Extracted (AVE) = Σλ² / number of indicators*

*Thresholds: CR>0.70 acceptable, >0.80 good; AVE>0.50 acceptable, >0.60 good*

**Invariance Testing Across Equipment Types**

**Multi-group CFA testing measurement invariance:**

| **Model** | **χ²** | **df** | **Δχ²** | **Δdf** | **p-value** | **ΔCFI** | **Interpretation** |
| --- | --- | --- | --- | --- | --- | --- | --- |
| Configural (same structure) | 284.5 | 153 | - | - | - | - | Baseline |
| Metric (equal loadings) | 297.3 | 171 | 12.8 | 18 | 0.80 | -0.003 | **Invariant** (loadings equivalent across groups) |
| Scalar (equal intercepts) | 318.7 | 189 | 21.4 | 18 | 0.26 | -0.007 | **Invariant** (intercepts equivalent across groups) |

*Equipment types: High-tech (n=125), Moderate-tech (n=142), Low-tech (n=80)*

*Interpretation: Full measurement invariance achieved (ΔCFI<0.01), indicating indicator meanings and scale properties are equivalent across different equipment types*

**Model Specification (Mplus Syntax Summary)**

MODEL:

Equipment BY EquipUtil* MaintResp PredictAcc MultiModal;

Operational BY DynAlloc* RealQual RoomTurn PatientFlow;

Staff BY WorkInteg* TechAdopt CommEffect ProfDev;

Equipment@1;

Operational@1;

Staff@1;

OUTPUT: STDYX MODINDICES (10) RESIDUAL;

**Note:** The confirmatory factor analysis provides strong empirical support for the hypothesized three-dimensional structure of the AI-enhanced quality model. Excellent model fit indices (CFI=0.96, RMSEA=0.048), strong factor loadings (range: 0.67-0.91, all p<0.001), appropriate inter-factor correlations (r=0.51-0.64), and superior fit compared to alternative structures demonstrate that the Equipment Management, Operational Efficiency, and Staff Performance dimensions are empirically valid, theoretically meaningful, and statistically distinct yet appropriately interrelated. The model demonstrates measurement invariance across equipment types and strong reliability and validity properties, supporting its use for comprehensive operating room quality assessment.
