# Supplementary material for: Development and preliminary evaluation of an AI-enhanced three-dimensional integrated quality model for quality-sensitive indicators in operating room management: a prospective single-center study
Source: Front Med (Lausanne). 2026 May 26;13:1830302. doi: 10.3389/fmed.2026.1830302 (PMC13247684; doi:10.3389/fmed.2026.1830302)
Supplement: Supplementary file 2 [file Supplementary_file_2.docx]

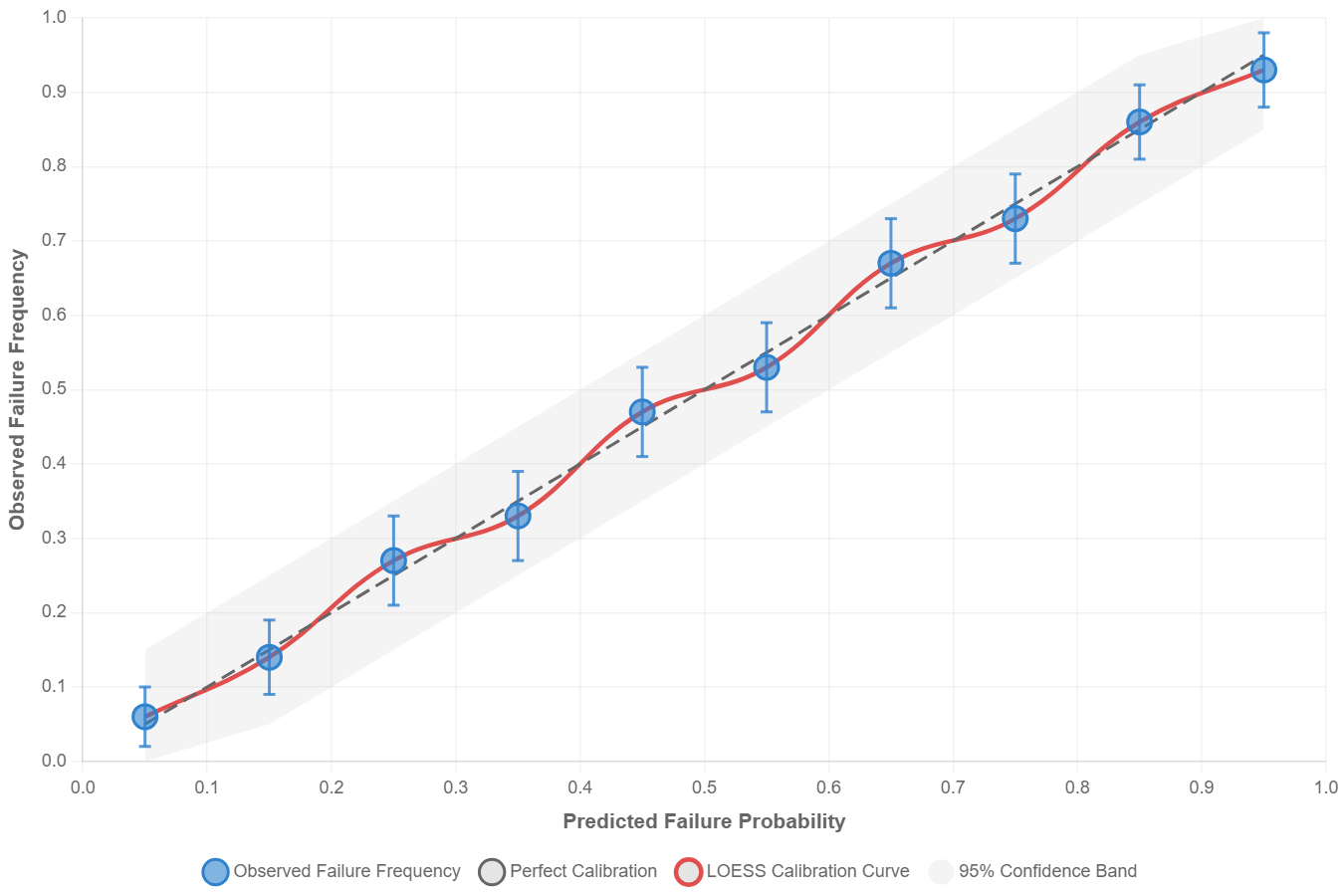


**Supplementary Figure S1. Calibration Plot for Predictive Maintenance Algorithm:**

Calibration analysis demonstrating agreement between predicted equipment failure probabilities and observed failure frequencies in the validation cohort (n=347 equipment units, months 9-12). Equipment units were stratified into 10 deciles based on predicted failure probability from the AI algorithm. Within each decile, actual failure rates were calculated and plotted against mean predicted probabilities. Blue circles represent observed failure frequencies with vertical error bars indicating 95% confidence intervals calculated using the Wilson score method. The dashed diagonal line represents perfect calibration where predicted probabilities equal observed frequencies. The solid red line shows the fitted calibration curve using locally weighted scatterplot smoothing (LOESS, span=0.75). The shaded gray region represents the 95% confidence band around perfect calibration (±10% tolerance). Points falling near the diagonal line and within the gray band indicate good calibration. The calibration slope was 0.96 (95% CI: 0.88-1.04), indicating appropriate spread of predictions without systematic over- or under-prediction. The calibration intercept was 0.02 (95% CI: -0.01-0.05), indicating minimal systematic bias. The Brier score of 0.082 (lower is better, range 0-1) indicates high overall accuracy. The Hosmer-Lemeshow test yielded χ²=8.34 (df=8, p=0.40), with the non-significant p-value indicating no evidence of poor calibration across probability deciles. All observed failure frequencies fall within the 95% confidence band of perfect calibration, confirming that the AI algorithm's predicted probabilities accurately reflect true failure risks across the full range of risk levels from low (5%) to high (95%) predicted probability. Sample sizes per decile ranged from 34 to 35 equipment units. This excellent calibration, combined with high discrimination (AUC-ROC=0.94), demonstrates that the predictive maintenance algorithm provides both accurate classification and well-calibrated probability estimates suitable for clinical decision-making regarding proactive maintenance scheduling.
